# Commensal Fungus Candida albicans Maintains a Long-Term Mutualistic Relationship with the Host To Modulate Gut Microbiota and Metabolism

**DOI:** 10.1128/spectrum.02462-22

**Published:** 2022-09-22

**Authors:** Doureradjou Peroumal, Satya Ranjan Sahu, Premlata Kumari, Bhabasha Gyanadeep Utkalaja, Narottam Acharya

**Affiliations:** a Laboratory of Genomic Instability and Diseases, Department of Infectious Disease Biology, Institute of Life Sciences, Bhubaneswar, India; b Regional Centre for Biotechnology, Faridabad, India; Institut Pasteur

**Keywords:** microbiome, mycobiome, diet, genomics, BMI, hormone, probiotics, candidiasis, obesity, gut microbiome, metabolism, metagenomics

## Abstract

Candida albicans survives as a commensal fungus in the gastrointestinal tract, and that its excessive growth causes infections in immunosuppressed individuals is widely accepted. However, any mutualistic relationship that may exist between C. albicans and the host remains undetermined. Here, we showed that a long-term feeding of C. albicans does not cause any noticeable infections in the mouse model. Our 16S and 18S ribosomal DNA (rDNA) sequence analyses suggested that C. albicans colonizes in the gut and modulates microbiome dynamics, which in turn mitigates high-fat-diet-induced uncontrolled body weight gain and metabolic hormonal imbalances. Interestingly, adding C. albicans to a nonobesogenic diet stimulated the appetite-regulated hormones and helped the mice maintain a healthy body weight. In concert, our results suggest a mutualism between C. albicans and the host, contrary to the notion that C. albicans is always an adversary and indicating it can instead be a bona fide admirable companion of the host. Finally, we discuss its potential translational implication as a probiotic, especially in obese people or people dependent on high-fat calorie intakes to manage obesity associated complications.

**IMPORTANCE** Candida albicans is mostly considered an opportunistic pathogen that causes fetal systemic infections. However, this study demonstrates that in its commensal state, it maintains a long-term mutualistic relationship with the host and regulates microbial dynamics in the gut and host physiology. Thus, we concluded that C. albicans is not always an adversary but rather can be a bona fide admirable companion of the host. More importantly, as several genomic knockout strains of C. albicans were shown to be avirulent, such candidate strains may be explored further as preferable probiotic isolates to control obesity.

## INTRODUCTION

Humans are colonized by a full array of microorganisms, popularly coined as microbiota, irrespective of being in either healthy or diseased states (1, 2). The human microbiota comprises bacteria, archaea, fungi, viruses, and a few protozoans. Bacteria by far are the most abundant microbes and are estimated to represent 3-fold more than the total number of somatic cells present in a human body. A large body of evidence suggests that the gut microbiota, especially bacteria, is the main biotic factor that regulates obesity, metabolic syndrome, type 2 diabetes (T2D), and the immunological and neurological status of its human host (3–5). The gut microbiota of healthy individuals is mostly composed of bacteria from the *Firmicutes* and *Bacteroidetes*. The proportion of these bacteria often gets altered in obese animals and humans. Apart from bacteria, humans harbor more than 66 genera and 184 species of fungi that comprise the human gut mycobiome, while *Candida*, *Saccharomyces*, and *Cladosporium* species predominate. Although no differences were detected in the richness of the mycobiome between nonobese and obese subjects, family diversity was significantly reduced in obese subjects compared to that in nonobese individuals (6). Thus, the mycobiome could play a role in modulating host physiology similar to and equally as important as gut bacteria.

*Candida* species are usually found on the mucosal surfaces of ~50 to 70% of healthy humans (7, 8). Although they survive as a commensal in healthy individuals, they have been mostly explored as opportunistic fungal pathogens. The overgrowth of *Candida* is considered to be a prerequisite for endogenous infection, with the gastrointestinal (GI) tract serving as a reservoir in the development of candidiasis (9, 10). The development of candidiasis has been linked to microbial dysbiosis and host immunity as causative factors, and candidiasis is accountable for the increased rate of mortality and morbidity, especially in immunosuppressed individuals. Among the *Candida* species, Candida albicans has been predominantly isolated and extensively studied. Although there are contrasting reports, it is thought that the overuse of broad-spectrum antibiotics causes dysbiosis to facilitate C. albicans growth (11, 12). One of the widely accepted rationales is that the killing of commensal bacteria vacates sites on host epithelial tissues for fungal adhesion and reduces competition for nutrients and other resources to promote C. albicans colonization and invasive growth. Reconciling with this, a recent report suggested that the β-lactam antibiotics while killing bacteria release a significant amount of peptidoglycan fragments that potentiate filamentation in C. albicans and systemic fungal dissemination. Contrarily, another report found that the depletion of bacteria alone in the gut is not sufficient to promote the invasive growth of C. albicans: additionally, it requires immunosuppression of the host and damage to the mucosal layer (13). Thus, it appears that both the commensal status and pathogenic status of C. albicans seem to be influenced by direct and indirect interactions with the gut microbiota.

A precise function of C. albicans as a commensal in the host has yet to be defined. Although some studies have suggested a link between expansion in *Candida* spp. and diabetes and inflammatory disorders of the gastrointestinal tract, with a possible active role of C. albicans, the difference in the relative abundance of C. albicans in obesity was not observed (14, 15). Contrarily, a recent study points out an association of C. albicans, Candida kefyr, and Rhodotorula mucilaginosa fungal species with human obesity (16). Therefore, in this study using animal models, we have explored a direct association between C. albicans and host physiology. Since in addition to maternal transmission, food ingestion is another way microorganisms enter the GI tract (17), we subjected BALB/c mice to consume C. albicans mixed diets and compared gut microbiota and metabolic changes with those in the control mice. Our findings suggested that the dietary C. albicans antagonizes the deleterious effects of a high-fat diet (HFD) and restores the alterations in metabolism induced by the obesogenic diet by changing the gut microbiota to a healthy standard. Interestingly, adding C. albicans to a nonobesogenic diet stimulated the appetite-regulated hormones and helped the mice to maintain a healthy body weight. Therefore, we concluded that C. albicans during its commensal state maintains a mutualistic relationship with the host.

## RESULTS

### Dietary C. albicans in the gut does not induce body weight gain while it protects mice from high-fat-diet-induced obesity.

**FIG 1:**
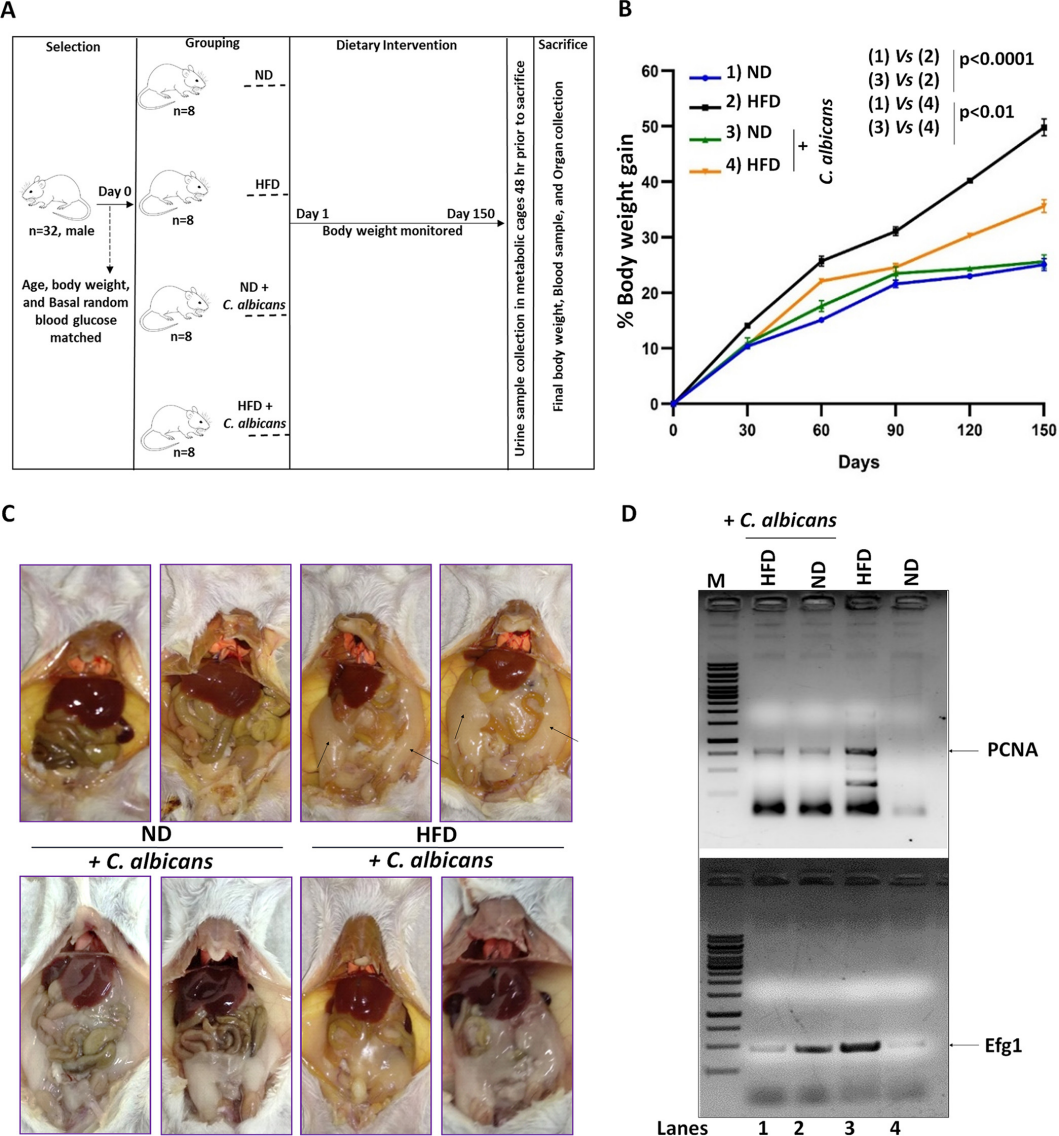
DOI-induced body weight gain, fat deposits, and fungal colonization in male BALB/c mice. (A) Schematic workflow of the study of the diet-induced obesity model and the role of C. albicans in weight gain in mice; (B) kinetics of percentage gain in body weight with respect to duration (corrected from basal body weight in grams) from mice fed with or without C. albicans on a normal diet (ND) or high-fat diet (HFD). Statistical significance was calculated using two-way ANOVA and Tukey’s multiple-comparison test (*, *P* ≤ 0.05; **, *P* ≤ 0.01; ***, *P* ≤ 0.001; ****, *P* ≤ 0.0001). (C) Representative images of mesenteric fat indicated by arrows from each group of mice under dissection are displayed. The 4 images above are from the respective diet without C. albicans, while the 4 images below are from the respective diet with C. albicans. (D) PCR amplification of CaPCNA and CaEfg1 ORFs from the metagenomic DNA isolated from fecal samples. Lanes: M, DNA ladder; 1, HFD-C. albicans; 2, ND-C. albicans; 3, HFD; 4, ND.

Since BALB/c mice are highly susceptible to C. albicans systemic infection (18), we considered this to be an ideal system to determine the effect of the dietary form of C. albicans on murine physiology and if any infections are caused thereafter. Thus, the study involved four groups of similarly housed male mice matched for age, body weight, and random blood glucose (RBG) (*n* = 8 per group on average). These mice were subjected to feeding on a normal diet (ND), high-fat diet (HFD), ND mixed with C. albicans, and HFD mixed with C. albicans over a period of 150 days (Fig. 1A). About 1 g (~10^10^ cells) of freshly grown cultured C. albicans cell pellet (optical density at 600 nm [OD_600_] = 1) was mixed with 100 g of powdered diet in a sterile environment, and the soft dough was prepared with sterile water. Similar consistency of food without the fungal mix was also prepared. About 5 g of food per mouse was provided daily in the morning, and on an average ~70 to 80% of food was consumed in each cage during the 24-h feeding period, again suggesting no limitation of the supplied food. This also indirectly implied that the supplementation of C. albicans had no or minimal effect on food intake by individual mice. On day 0, the body weight and random blood glucose of mice in each group ranged from 23.1 ± 0.5 to 24.4 ± 0.6 g and 145 ± 16 to 152 ± 16 mg/dL, respectively (see Table S1 in the supplemental material). Up to 90 days of dietary intervention, the percentages of body weight gain were similar in each group and were consistent with the previously reported data from the BALB/c diet-induced obesity (DIO) model, suggesting the use of similar experimental conditions among these studies (19–21) (Fig. 1B). Subsequently, the percentage of body weight doubled after every 8 weeks of HFD consumption alone, with an average body weight of ~36 g on the 150th day, an increase of ~50% compared (*P ≤ *0.0001) to the normal-chow-fed group of mice that gained only ~25% for the period over their initial weight (Fig. 1B and Fig. S1A). However, the presence of C. albicans culture in the diets had an opposing influence on body weight gain of normal-chow- versus HFD-fed mice. Interestingly, while the presence of C. albicans improved the normal-chow-fed mice in gaining weight marginally (*P ≤ *0.01), it considerably reduced the excessive body weight gain in HFD-fed mice. Only an increase in body weight gain of ~35% was found in mice subjected to the HFD mixed with *C. albicans*. In addition to an increase in body weight, the presence of visceral and epididymal fat which is implicated in adiposity, was observed maximally in the abdomen of HFD-fed mice compared to the other groups at 150 days of feeding (Fig. 1C). The HFD-*C. albicans*-fed group displayed relatively less visceral fat depositions. Though the ND-fed mice preserved a negligible amount of abdominal fat, interestingly the ND-*C. albicans*-fed group had relatively a higher deposition of epididymal fat (Fig. 1C). Thus, these results signify a possible beneficial effect of C. albicans on host metabolic status impacting obesity and metabolic syndrome depending on the dietary fat level.

### Colonization of dietary C. albicans in the murine gut.

Generally, gut and feces share similar microbial populations (22). To understand whether the differential body weight gain in various groups of mice is due to gut colonization of C. albicans, fecal metagenomic DNA was isolated and the presence of C. albicans was confirmed by both endpoint and real-time PCRs using specific primers of *C. albicans* PCNA (CaPCNA) (a DNA clamp protein involved in replication) and CaEfg1 (a transcription factor involved in filamentation) (23–25). Interestingly, amplicons of both of the open reading frames (ORFs) were detected in the meta-DNA of mice subjected to a normal diet, suggesting that the mouse gut is a natural habitat of C. albicans, albeit with less abundance (Fig. 1D, lane 4). Interestingly, the amount of PCR product was detected to be greater in HFD-fed mice than in mice fed the mix containing HFD/ND+C. albicans (compare lane 3 with lanes 1 and 2), indicating counteractive effects of dietary compositions and C. albicans gut colonization. While HFD alone facilitated excessive growth of GI-resident C. albicans, dietary C. albicans regulated its own colonization in HFD/ND-C. albicans mix-fed mice. Real-time PCR analyses further confirmed greater abundance of C. albicans in the mice fed on fungus mix-regular diet and HFD alone in comparison to the other two groups of mice (Fig. S1B). A higher threshold cycle (*C_T_*) value suggests a lesser presence of that particular species and the control-diet- and fungal mix-HFD-fed mice exhibited the least colonization by C. albicans in the gut. Next, the presence of C. albicans in the gut was confirmed by estimating the CFU of various fecal samples and their confirmation by colony PCR (Fig. S1C). Since these groups of mice were fed with the same diet composition over a period of 150 days, it is very unlikely that the presence of C. albicans in fecal samples is temporal. Moreover, the presence of C. albicans in the fecal samples of ND-fed mice again suggested that this fungus is a natural colonizer in the murine gut.

### HFD and C. albicans differentially regulate gut microbiota.

**FIG 2:**
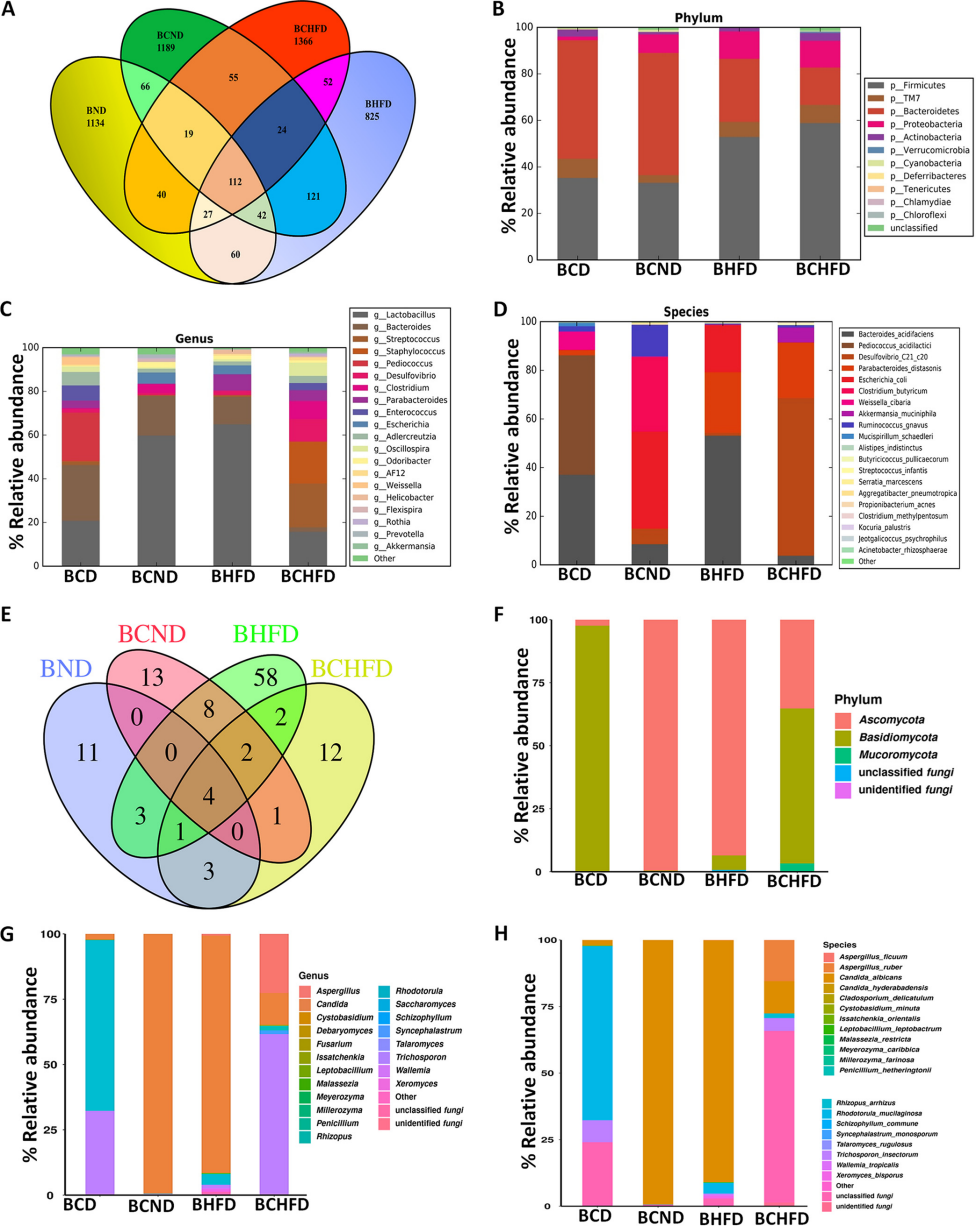
16S and 18S rDNA metagenomic analyses. (A) Venn diagram depicting unique and shared OTUs among the four cohorts. Taxonomic classification was carried out, and assignments were made into the top 10 phyla (B), top 20 genera (C), and top 20 species (D). (E) Venn diagram depicting unique and shared ASVs among the four cohorts. Taxonomic classification was carried out, and assignments were made into the top 10 phyla (F), top 20 genera (G), and top 20 species (H). BND, metagenomic DNA isolated from fecal samples from BALB/c mice fed the normal diet; BCND, metagenomic DNA isolated from fecal samples from BALB/c mice fed the normal diet with C. albicans mix; BHFD, metagenomic DNA isolated from fecal samples from BALB/c mice fed the high-fat diet; BCHFD, metagenomic DNA isolated from fecal samples from BALB/c mice fed the normal diet with C. albicans mix.

Since dysbiosis in the GI tract is one of the causative factors responsible for excessive growth of and infections by *C. albicans*, and because of our observations of the differential effect of diet and dietary C. albicans on gut colonization of C. albicans (Fig. 1D), for a broader perspective we wanted to monitor the effect of a diet and exogenously added C. albicans on the diversity and richness of gut microbiota. As the gut is mostly filled with bacteria and fungi, we used the genomics approach to identify various microbes by sequencing 16S ribosomal DNA (rDNA) and the internal transcribed spacer (ITS) region of 18S rDNA from fecal meta-DNA samples (Fig. 2 and Fig. S2). Our 16S rDNA analyses revealed an abundance of 1,263 to 1,695 bacterial species in all four samples (Table S2). HFD-fed mice showed poor microbial richness (1,263 operational taxonomic units [OTUs]), however, mixing of dietary C. albicans with HFD restored the microbial composition to that similar to that of mice fed with normal diets (1,500 to 1,695 OTUs). An alpha rarefaction plot that helps in comparing diversity across samples also indicated poor richness of microbial species in HFD-fed mice (Fig. S3A). Alpha rarefaction plots were constructed by using Mothur, whereas the Simpson, Shannon, and Chao1 diversity indices were used to generate alpha rarefaction curves. Thus, while HFD induced dysbiosis, dietary C. albicans had a positive influence on gut microbial diversity. Among the four samples, only 112 bacterial species were found to be common, and 1,134, 1,189, 825, and 1,366 species were specifically detected in the guts of mice fed with ND, ND-C. albicans, HFD, and HFD-C. albicans, respectively (Fig. 2A). Less than 1% of sequences corresponding to unclassified or unknown bacteria were detected in each sample. The proportion of bacteria from *Firmicutes* and *Bacteroidetes* contribute maximally to healthy gut microbiota. Obese individuals were reported to possess relatively decreased *Bacteroidetes* and increased *Firmicutes* in their guts compared to lean subjects, and in this study also, a similar trend was observed involving BALB/c mice as a model organism when they were subjected to HFD. Interestingly, although the alteration in richness proportion of these two bacterial phyla was quite apparent between normal- and high-fat diet samples, we found that the percentages of *Firmicutes* (~33 to 35% in the normal-diet sample versus 53 to 59% in the HFD sample) and *Bacteroidetes* (~51 to 53% in the normal-diet sample versus 16 to 27% in the HFD sample) among the samples were not altered significantly by the dietary intervention of C. albicans (Fig. 2B). It appeared that HFD had a more pronounced effect on the proportion of *Firmicutes* and *Bacteroidetes* in the population than the addition of dietary C. albicans. In addition to these two phyla, members of the *Proteobacteria* were the third-most-abundant species, and they were mostly enriched in all the samples (~8 to 11%) except in fecal samples from mice fed the normal diet, where they were poorly detected (~1%). Thus, *Proteobacteria* could help in gaining healthy body weight. Furthermore, the OTUs were analyzed to distribute them in the top 20 classes, orders, and families (Fig. S3B, C, and D). At the genus and species levels, the addition of fungal mix to the diet modulated the bacterial composition drastically (Fig. 2C and D). For example, *Lactobacillus* species were highly abundant in HFD samples but reduced by the addition of C. albicans (64% to 15%), and the effect was reversed in normal-diet samples (increased from 20% to 60% with dietary *Candida*). Our genomics analyses also revealed the abundance of diet-specific bacteria. The normal-diet-fed mice only possessed *Pedicococcus* sp., while Streptococcus and Staphylococcus species were only detected in mice fed the mixed fungus-high-fat diet (Table S3).

Although there is a differential opinion on selecting the internal transcribed spacer 1 (ITS1) or ITS2 region of the eukaryotic ribosomal cluster for fungal species identification by high-throughput sequencing, both regions are used quite frequently (26, 27). For this study, ITS1 region-specific primers were used. The ITS1 region is recognized as a suitable barcode region for species-level identification of fungal organisms and has the characteristics to allow wide taxonomic coverage (28). The ITS-based sequencing yielded 205,305 to 267,408 sequence reads, of which 47,840 to 96,481 corresponded to 118 amplicon sequence variants (ASVs) belonging to 3 different phyla, 11 classes, 18 orders, 28 families, and 39 different genera of fungi (Fig. 2E to H and Table S2). Fungal sequences were detected in every sample analyzed, and only <1% of sequences corresponded to unclassified or unknown fungi. Interestingly, only 4 ASVs were common to all the four samples sequenced, and according to the alpha diversity index plot, the DNA sample of the HFD+C. albicans group showed the maximum variation (Fig. 2E and Fig. S4A). QiiME was used to calculate alpha diversity indices, whereas the Simpson, Shannon, and Chao1 diversity indices were used to generate alpha rarefaction curves. Fungi belonging to the phyla Ascomycota and Basidiomycota were abundant in all the samples. Fungal species belonging to the phylum Basidiomycota (97.6%) were enriched in normal-diet-fed mouse gut, whereas HFD-fed or mixed C. albicans-diet-fed mice showed an abundance of fungi belonging to Ascomycota (~93.5 to 99.5%). Interestingly, the presence of C. albicans in the HFD alters the ratio of Ascomycota and Basidiomycota from 16:1 (93.54% versus 5.74%) to 0.6:1 (35.25% versus 61.51%) in the gut; also, fungi belonging to the Mucoromycota got enriched (Fig. 2F). While species belonging to the classes Saccharomycetes, Sordariomycetes, and Tremellomycetes were commonly found in all the samples, dietary C. albicans suppressed enrichment of the classes Cystobasidiomycetes, Dothideomycetes, and Microbotryomycetes. The abundance of Eurotiomycetes and Mucoromycota was only found in HFD-fed mouse samples, but C. albicans addition to the food reduced their composition significantly (Fig. S4B, C, and D). At the genus level, *Candida* and *Trichosporon* fungi were commonly found in all the samples, whereas Aspergillus and Fusarium were observed in all the samples except the ND group. Additionally, we also observed a diet-specific abundance of the fungal genus (Fig. 2G and H). While in the normal-diet-fed mice only the fungi *Monographella*, *Neoascochyta*, *Papiliotrema*, and *Wickerhamomyces* were enriched, species belonging to the genera *Acremonium*, *Cladosporium*, *Clavispora*, *Didymella*, *Leptobacillium*, *Issatchenkia*, *Microascus*, *Millerozyma*, *Myceliophthora*, *Ovatospora*, *Penicillium*, *Saccharomyces*, *Starmerella*, *Talaromyces*, and *Xerochrysium* were seen in mice subjected to the HFD. Similarly, the genera *Naganishia*, *Schizophyllum*, and *Sterigmatomyces* and *Mucor*, *Rhizopus*, and S*yncephalastrum* were abundant in mice fed the mixed C. albicans-normal and -high-calorie-content diets, respectively (Table S3). Consistent with the genus level, C. albicans species were the standout species in all the samples. Other species, such as C. dubliniensis, *C. hyderabadensis*, and *C. sake*, were also observed in few samples. However, we did not detect other *Candida* species prevalent in humans, such as C. tropicalis, C. parapsilosis, C. glabrata, C. krusei, and C. lusitaniae (6, 29). An experimental animal model of colitis demonstrated the abundance of C. tropicalis in the fecal DNA of C57BL/6 mice (30). So, whether the gut of a specific strain of animal favors colonization of certain species of *Candida* requires further investigation. As shown in Fig. 1D as well, a low but significant abundance (~2%) of C. albicans was found in the metagenome of normal-died-fed mice, but the addition of dietary C. albicans to the normal diet or HFD alone facilitated its better colonization (~90 to 99%). Surprisingly, only a marginal increase in the colonization of C. albicans (12%) in the gut of mixed C. albicans-HFD-fed mice was noticed. This result suggests that while HFD by itself facilitates C. albicans growth in the gut, the presence of dietary C. albicans somehow negates the effect of HFD on its own colonization. In terms of fungal species composition, the HFD-induced metagenome is highly diversified, and 28 different species of fungus were seen. However, the addition of C. albicans to HFD reduced the species diversity drastically to only 10 different species. Fungal species diversity remained low in the normal-diet-fed mice irrespective of dietary C. albicans addition. Rhodotorula mucilaginosa and Trichosporon insectorum were the highly enriched species in the metagenome of the control-diet-fed mice. Aspergillus *ruber* and Rhizopus arrhizus are the two notable fungal species that got specifically enriched by the addition of dietary C. albicans to HFD.

From our genomics analyses, we conclude that HFD reduces bacterial diversity but increases fungal composition, and dietary C. albicans colonizes in the mice and regulates its own colonization in the host consuming a high-fat diet, in addition to regulating the abundance of other microbial species. The abundance of C. albicans in regular-chow- and HFD-fed mice suggests that C. albicans is a critical contributor to the mouse’s gut microbiota. Since alternations in the gut microbiota have been shown to influence the metabolites released by bacterial and fungal species that are known to impact host physiology (31–33), C. albicans by being a commensal and by maintaining eubiosis could play an important role in modulating host physiology, and thus it warrants further investigation.

### Dietary C. albicans reduced adiposity-associated hormones in mice with diet-induced obesity.

**FIG 3:**
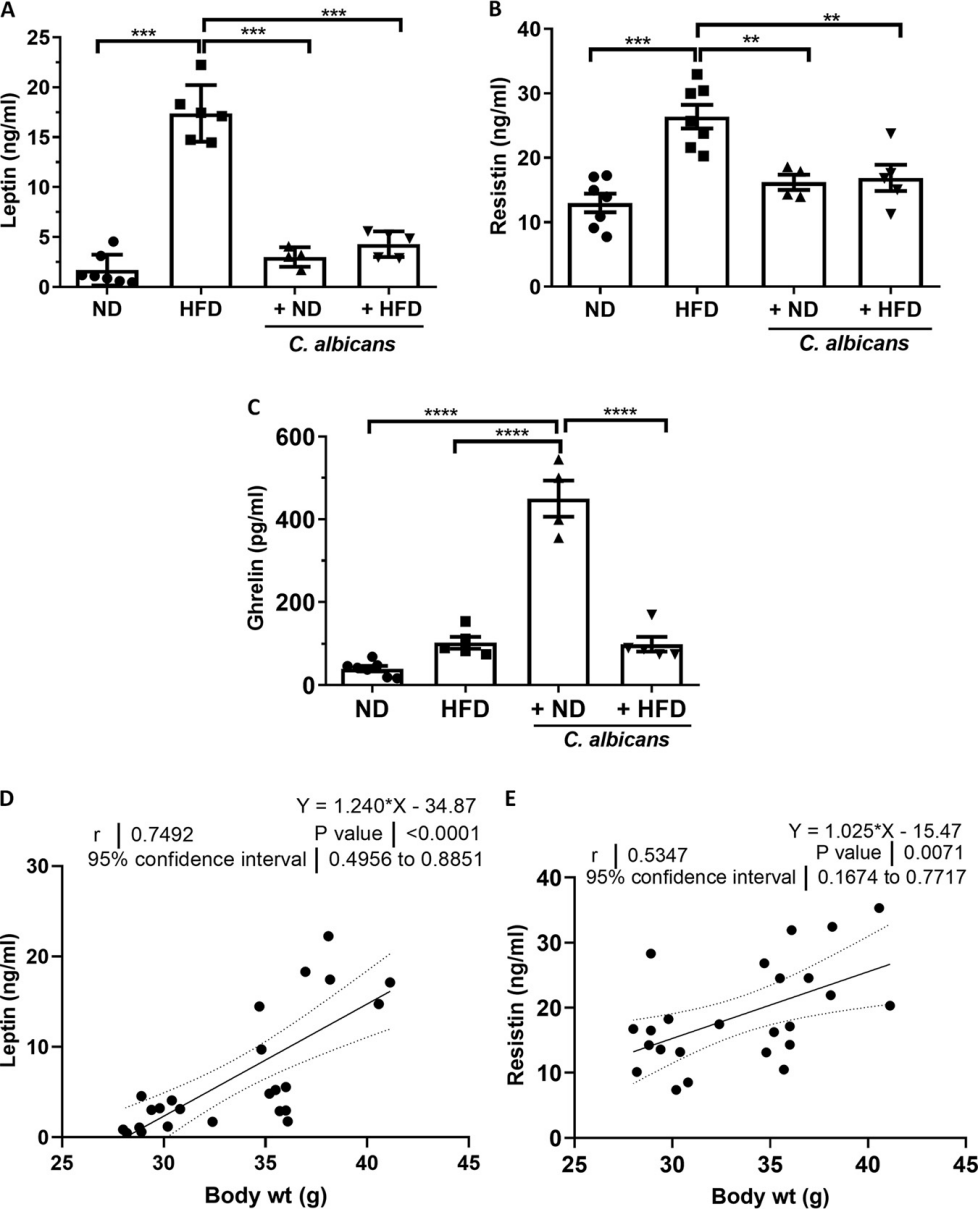
Diet- and dietary C. albicans-induced hormonal changes associated with body weight. Results are the mean and standard error of the mean from plasma (A) leptin, (B) resistin, and (C) ghrelin levels from each of four groups of mice. Statistical significance was calculated using one-way ANOVA and Tukey’s multiple-comparison test (*, *P* ≤ 0.05; **, *P* ≤ 0.01; ***, *P* ≤ 0.001; ****, *P* ≤ 0.0001). (D and E) Regression and Person’s correlation analysis of body weight versus the levels of (D) leptin and (E) resistin from individual mice irrespective of dietary intervention.

Leptin and resistin play important roles in obesity and type II diabetes (T2D), and together with gastrointestinal ghrelin they regulate energy balance and body weight (34, 35). DIO and genetic models of obesity reported increased leptin and resistin levels. Our estimation revealed that plasma leptin (14 to 20 ng/mL; *P ≤ *0.001) and resistin (20 to 32 ng/mL; *P ≤ *0.001) levels in HFD-fed mice were ~7- to 10-fold and ~1.8- to 2.5-fold higher than their respective levels in the normal-diet-fed mice (1 to 3 ng/mL and 7.7 to 17.2 ng/mL) (Fig. 3A and B). Interestingly, C. albicans in HFD significantly reduced both leptin 4- to 6-fold (2.5 to 5.5 ng/mL; *P* < 0.001) and resistin 1.5- to 1.7 (13.9 to 18.5 ng/mL; *P ≤ *0.01) compared to HFD fed alone. However, leptin and resistin levels were found to be similar in mice subjected to a diet mixed with fungus regardless of calorie content, suggesting a direct or indirect role of dietary C. albicans in controlling the hormones. Furthermore, a linear regression analysis revealed a strong positive correlation of body weight gain (grams) versus the level (nanograms per milliliter) of leptin (*r* = 0.749; *P* ≤ 0.0001) or resistin (*r* = 0.534; *P* ≤ 0.007) in each mouse irrespective of treatment background (Fig. 3D and E). The hormone ghrelin stimulates appetite, increases food intake, and promotes fat storage in the reproductive system. Levels of acylated ghrelin (active ghrelin) in plasma of groups fed ND (16.5 to 68.1 pg/mL), HFD (74.5 to 153.5 pg/mL), and HFD-C. albicans (74.57 to 169 pg/mL) were not statistically different but markedly increased by ~16-fold in the group of mice fed the mixed C. albicans and ND (355 to 544 pg/mL; *P ≤ *0.0001) (Fig. 3C). Thus, an increased active ghrelin level, along with epididymal fat deposition without changing body weight (Fig. 1C) in mice fed mixed C. albicans plus ND indicated the possible role of dietary C. albicans in host metabolic capacity, in addition to increasing feeding tendency by maintaining hunger sense. Furthermore, estimation of the leptin/ghrelin ratio as a predictor of body mass index (BMI) as reported earlier in human studies (36, 37) was found to be significantly higher in HFD-fed mice (~7-fold high) than in ND-fed mice, but it was significantly reduced to ~3.5-fold by C. albicans dietary intervention. The C. albicans addition to ND did not affect the leptin/ghrelin ratio (Fig. S5A). These results strongly suggest that HFD alone induced the onset of obesity in BALB/c mice, but dietary C. albicans inhibited the process.

### Altered hormones of energy metabolism in DIO were restored upon feeding C. albicans in a diet.

**FIG 4:**
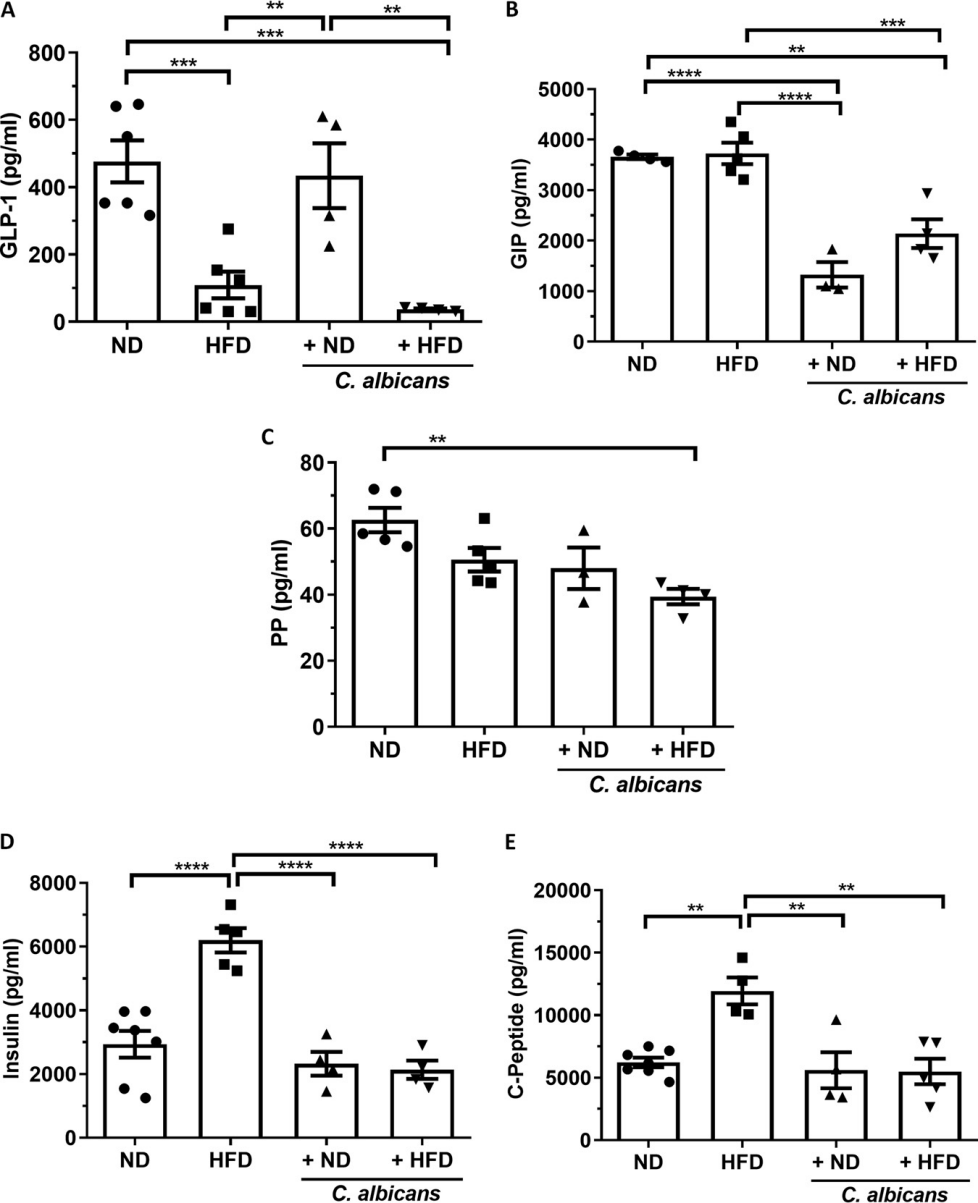
Key metabolic hormonal imbalance associated with energy metabolism and diets. Results are the mean and standard error of the mean from various metabolic hormones from blood from each of the groups of mice on the 150th day (~22 weeks) of dietary intervention. Statistical significance was calculated using one-way ANOVA and Tukey’s multiple-comparison test (*, *P* ≤ 0.05; **, *P* ≤ 0.01; ***, *P* ≤ 0.001; ****, *P* ≤ 0.0001). (A) Glucagon-like peptide-1 (GLP-1) level; (B) glucose-dependent insulinotropic polypeptide (GIP) level; (C) pancreatic polypeptide (PP); (D) insulin; (E) C-peptide.

The roles of gastrointestinal (GI) peptide hormones in the regulation of GI function and energy balance have been demonstrated (38, 39). In contrast to ghrelin, a low level of glucagon-like peptide 1 (GLP-1) stimulates more eating, thereby increasing the probability of onset of obesity. GLP-1 was significantly high (0.3 to 0.7 ng/mL; *P ≤ *0.001) in the ND-fed mice irrespective of C. albicans presence, suggesting them to be low-appetite groups. Contrastingly, GLP-1 (~0.1 ng/mL) was significantly low in the HFD group, indicating a high tendency to gain more weight (Fig. 4A). Like GLP-1, glucose-dependent insulinotropic polypeptide (GIP) is also an incretin that is secreted in the proximal small intestine in the presence of nutrients in the gut and affects insulin secretion in response to a meal. GIP is also involved in lipid metabolism and is thought to promote fat deposition. GIP levels were elevated in the groups of mice fed a normal diet and obesogenic diet (3 to 4.5 ng/mL; *P ≤ *0.0001), and they were greatly reduced when the feed was blended with C. albicans culture (1 to 2 ng/mL) (Fig. 4B). Pancreatic polypeptide (PP) is secreted by PP cells of the pancreas, and its level is reduced under conditions associated with increased food intake but elevated in anorexia nervosa. On fasting, the plasma PP concentration is 80 pg/mL, and after the meal, it rises by 8 to 10 times (40). The plasma PP levels were significantly reduced in the HFD-C. albicans diet (~40 pg/mL; *P ≤ *0.01) in comparison to other groups of mice (~60 pg/mL) (Fig. 4C). However, the concentration of peptide tyrosine tyrosine (PYY), an anorexigenic peptide released from the cells in the ileum and colon, did not alter in any cohorts (~200 to 300 pg/mL) (Fig. S5B). C-peptide, insulin, glucagon, and amylin are released from the pancreas and regulate plasma glucose homeostasis. Insulin and C-peptide levels (~6 ng/mL and 12 ng/mL, respectively) were very high in the blood of mice subjected to an obesogenic HFD diet, and thus they appeared to be insulin resistant and hyperglycemic, but the presence of C. albicans in food reduced them to the respective standard levels (~2 ng/mL and 5 ng/mL) (Fig. 4D and E). The amylin and glucagon levels did not alter much in any of the experimental setups used, and they remained constant at 100 to 150 pg/mL of blood (Fig. S5C and D). A direct correlation between leptin and insulin released was also observed (Fig. S5E and F). These results suggested that the addition of C. albicans to the regular diet increases the level of the hunger hormone ghrelin but reduces GIP, the appetite-inducing hormone; thereby it helps to maintain body weight and prevents becoming overweight. Rising levels of insulin and C-peptide levels in HFD-fed mice and their restoration by the presence of dietary C. albicans suggested that this commensal fungus is just not a passive component of the gut but plays an active role in regulating metabolic conditions that predispose to obesity and diabetes.

### Dietary C. albicans suppresses diet-induced alteration in glucose and lipid metabolism.

**FIG 5:**
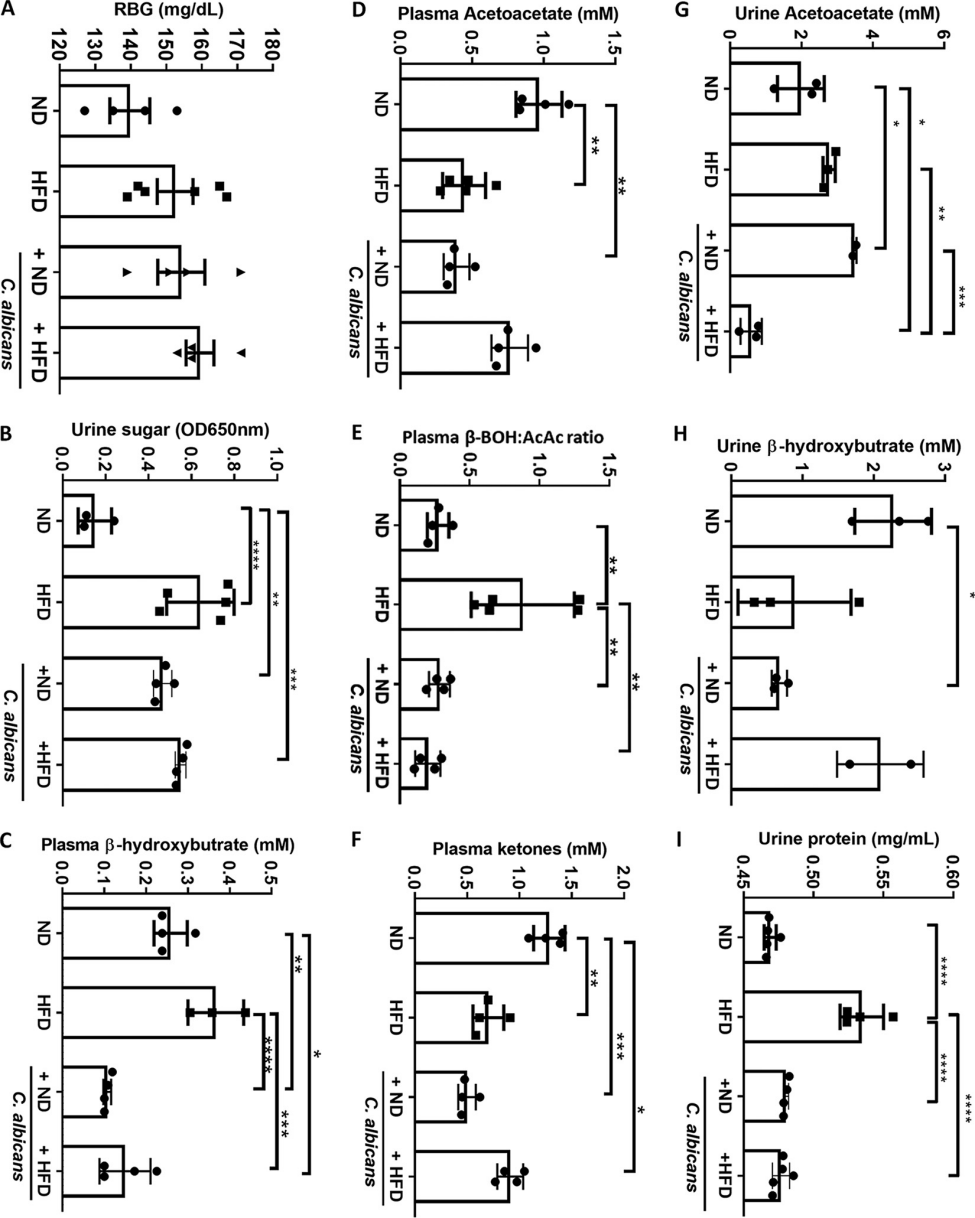
Diet-mediated alterations in metabolism. Shown are the mean and standard error from the (A) random blood glucose level, (B) urine sugar, (C) plasma β-hydroxybutyrate, (D) plasma acetoacetate, (E) β-hydroxybutyrate/acetoacetate ratio, (F) plasma ketones, (G) urine acetoacetate, (H) urine β-hydroxybutyrate, and (I) urine proteins of each mouse from the 4 groups of mice. Statistical significance was calculated using one-way ANOVA and Tukey’s multiple-comparison test (*, *P* ≤ 0.05; **, *P* ≤ 0.01; ***, *P* ≤ 0.001; ****, *P* ≤ 0.0001).

Next, we estimated random blood glucose (RBG) by using the GlucoOne blood glucose monitoring system and urine sugar by using Benedict’s solution reaction to determine any alterations in glucose metabolism in these mice. RBG levels in the range of 80 to 140 mg/dL before food and 100 to 160 mg/dL after taking food are considered to be normal. On the 150th day, RBG levels were highly variable among the animals irrespective of their diet intakes, and they varied from 125 mg/dL to 170 mg/dL (Fig. 5A). In comparison to day 0, on the 150th day the average RBG levels slightly increased from ~145 to 153 to ~158 to 177 (Table S1). While no traces of glucose were observed in the urine of normal-chow-fed mice, in the HFD-fed group, Benedict’s solution turned instantly green with the addition of urine, indicating the presence of a high level of reducing sugars (Fig. 5B and Fig. S6A). The bluish-green coloration of urine of mice fed with any diet but mixed with C. albicans culture suggested the presence of traces of sugars in their urine samples. Although we did not find a change in the sugar levels in the blood of various mouse groups, excess sugar in the urine of HFD-fed mice indirectly indicated a high level of sugars in the blood of those mice, which was reduced by supplementing the diet with C. albicans.

Fatty acids from adipose tissue or a high-fat diet are metabolized in the liver. Under certain circumstances, such as carbohydrate-restrictive diets, starvation, prolonged intense exercise, alcoholism, low food intake, insulin resistance, or diabetes mellitus, the liver produces ketone bodies by utilizing fatty acids (41). Acetoacetate, β-hydroxybutyrate (BOH), and acetone are water-soluble molecules collectively called ketone bodies. Here, we profiled urinary and plasma ketones to identify HFD- and *Candida*-induced changes in lipid metabolism. The level of plasma BOH was reduced to almost half (0.1 to 0.15 mM; *P*≤ 0.0001) in the mice fed with any diet when blended with C. albicans. In HFD-fed mice, the amounts of BOH were determined to be 0.25 to 0.35 mM (Fig. 5C). In addition, we also found a significant increase in the BOH/acetoacetate ratio in HFD that reflects higher BOH levels in the plasma of HFD-fed mice (Fig. 5C and E). Surprisingly, the urine excretion of BOH did not reflect the plasma level, indicating its possible utilization in these mice; however, it was 10-fold higher than that in the plasma. The urine BOH levels were high in the mice fed the normal and mixed C. albicans-high-fat diets compared to the other two groups of mice (Fig. 5H). The amounts of plasma total ketones and acetoacetate in ND-fed mice were found to be 1 to 1.25 mM, and the amounts were significantly reduced in mice fed HFD and diet with fungal mix (0.5 to 0.75 mM; *P* ≤ 0.001) (Fig. 5D and F). In general, ketones are not present in urine but appear when fats are burned for energy. About 2 to 3 mM acetoacetate was estimated in the urine of HFD-fed mice, and the amount was drastically reduced to 0.5 mM when the mice were allowed to eat the mixed fungus-HFD feed. About 2-fold more urine acetoacetate was observed in mice fed ND with the fungal mix than in those fed ND alone (Fig. 5G). These results indicated alterations in glucose and lipid metabolism in HFD-fed mice, which were restored upon adding the C. albicans mix. A high level of plasma BOH is independently associated with cardiovascular events and all causes of death in patients undergoing hemodialysis (42), and the risk seems to be reversed in the presence of C. albicans in mice fed the HFD diet.

### Renal functions are altered by HFD but not affected by C. albicans feeding.

The kidney plays an important role in maintaining metabolic homeostasis through urinary excretion of unwanted and excessive water-soluble metabolites. HFD was reported to induce renal injury (43). Since the kidney is the primary organ for invasive candidiasis, we evaluated the impact of HFD and dietary C. albicans on renal functions as well as on any systemic dissemination of C. albicans from the gut. Although the mice fed the fungal mixed diet were healthy, and none showed noticeable symptoms of any infections, the kidney size marginally increased in animals feeding on a fungal mixed diet. An average kidney size in mice on the regular diet was 0.28 g, and it increased maximally to 0.35 g in mice fed with the mixed fungus-HFD (Fig. S6B). To find out any relationship between kidney size and fungal load, the presence of C. albicans in kidney cross sections was examined by periodic acid-Schiff (PAS) staining. Interestingly, despite 150 days of fungal diet interventions, none of the kidney cross sections showed the presence of C. albicans, suggesting that the marginal increase in the size of kidneys in mice fed with fungus-supplemented chow was not due to the fungal load but rather could be due to their overall body weight or high cellular infiltrations in the kidney (Fig. S6C). Also, our results suggested that the impacts of dietary C. albicans and intravenous challenge of C. albicans in mice are very different, and dietary C. albicans does not disseminate into the bloodstream to cause any infection. The presence of a urinary protein indicates compromised renal function. We found more proteinuria in the mice fed with HFD (0.53 mg/mL; *P ≤ *0.0001) than all other groups of mice (~0.47 mg/mL) (Fig. 5I). Interestingly the urinary protein level in the dietary mixed C. albicans-HFD group was comparable to that in the group fed normal chow without or with fungal mix, suggesting that the presence of C. albicans in diet improved kidney function in the HFD group. More importantly, our study also suggested that the gut colonization of C. albicans induced by HFD or dietary C. albicans is mostly noninvasive.

## DISCUSSION

**FIG 6:**
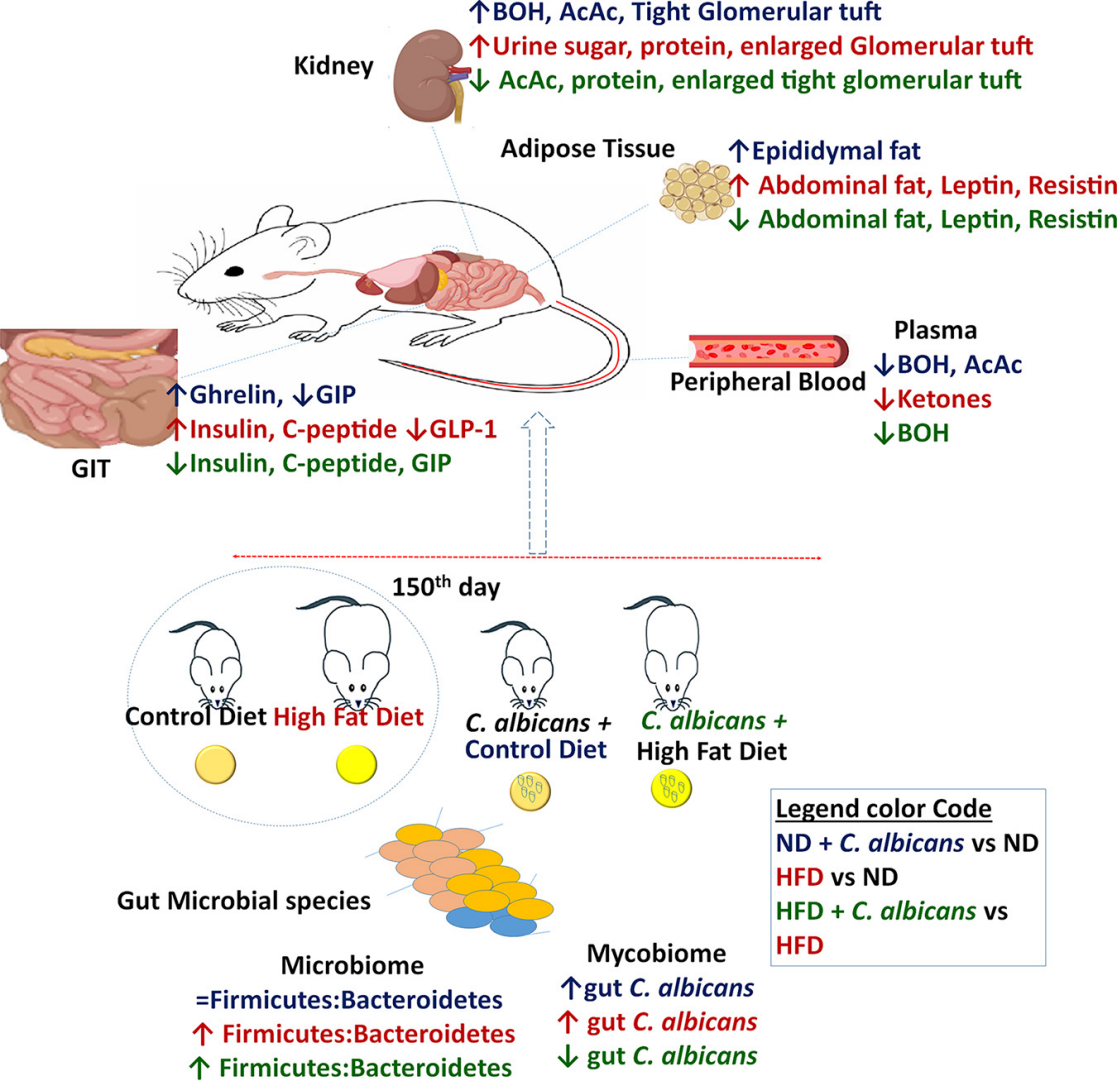
Overview of differential effect of dietary C. albicans on mice. Shown is a brief summary of the alternation in different metabolic parameters and microbiome in mice subjected to diets with different calorie contents without and with C. albicans probiotic. A legend for the color coding is provided in a blue box.

For decades, it has been known that C. albicans has no known terrestrial life cycle: it only survives and coexists both in humans and animals as a commensal organism. Several studies have been carried out considering it only as pathogenic, yeast but any beneficial symbiotic relationship that may exist between the host and the fungi had not been explored until the present study. A cooperative interaction between the microbiome and its hosts is critical in modulating host defense, metabolism, and reproduction (5, 31, 44, 45). Distinct microbial compositions were found in lean and obese subjects, although limited studies are available to decipher the precise role of individual bacterial and fungal species in the host’s functions. Many reports have suggested that C. albicans gut load is associated with obesity, regardless of other microbes’ involvement (16, 46, 47). Interestingly a diet that controls *Candida* growth in the gut, the “anti-*Candida* diet,” has also been on prescription for preventing obesity without any clear piece of evidence that supports the practice (48–51). Importantly, since one of the sources of gut microbe load has been diet, by using BALB/c mice as the model organisms, here we have attempted to understand the role of dietary C. albicans in its colonization, its influence on gut microbial composition, and obesity and associated metabolic changes. Several groups have shown that about 12 to 22 weeks of high-calorie food consumption results in ~40 to 80% body weight gain and promoted obesity in various strains of mice (52, 53). Herein, we found that BALB/c mice on HFD consumption gained ~50% of their original body weight in about 22 weeks, which is 2-fold higher than the mice fed normal chow for the same duration. Thus, we argued that the BALB/c strain is not a DIO-resistant strain as previously reported, only it takes a longer feeding duration (22 weeks) to gain body weight than other mouse strains (12 to 16 weeks). Nevertheless, despite differences in kinetics, most of the mice strains develop obesity by continuous feeding on a high-calorie diet. Interestingly and contrary to the practice of anti-*Candida* diet use, the presence of C. albicans in the diet could reduce excessive body weight gain: the effect was not either due to infection as the mice were active and healthy or due to low food intake. Th1 immunity is activated during colonization of C. albicans in mice, and the development of acquired resistance is associated with strong Th1 responses (54). Although BALB/c mice are susceptible to disseminated systemic candidiasis as they are Th2-biased strain (18), dietary C. albicans did not cause any noticeable infection irrespective of the types of diets consumed by the mice. This also indicates that overgrowth of C. albicans is not sufficient to cause fungal infection, and it may require some host factors, such as immunosuppression of the host and damage to the mucosal layer (55, 56). A summary of our findings related to C. albicans modulation of various parameters of obesity-related pathological disorders in mice is provided in Fig. 6.

Differential gut microbial composition in healthy versus obese animals and humans has been demonstrated, and here we have demonstrated further change due to the exogenous addition of C. albicans in the diet. The gut microbiota transiently changes depending upon a certain kind of diet, but we ensured the changes in gut microbiota in our model systems are stable by constantly providing the same diet for a longer period of duration so that the effect of dietary C. albicans can be determined. Interestingly, despite pooling the fecal samples of four groups of mice, the genome analyses obtained here were very similar to those reported earlier for individual mice or animal-specific metadata (17, 52, 53). Like in obese humans, this study also found similar results where the proportion of *Firmicutes* and *Bacteroidetes* is associated with obesity (57). While the abundance of *Akkermansia* (phylum *Verrucomicrobia*) is negatively associated with percentage of body fat growth in humans, relative abundances of *Lactococcus* from the phylum *Firmicutes* and with the genus *Allobaculum* (phylum *Bacteroidetes*) are positively associated with obesity (58). We did not detect *Lactoococcus* and *Allobaculum*, but rather Parabacteroides distasonis as a bacterial species positively associated with obesity. Our study revealed enrichment of Akkermansia muciniphila in the gut of mice subjected to mixed C. albicans-HFD only, suggesting its role in healthy body weight maintenance and energy expenditure. In addition to bacterial composition, the gut mycobiota also changed in these groups of mice. This study reports a detailed mycobiome analysis in animal models, and we clearly demonstrated C. albicans enrichment in HFD-fed mice as opposed to the normal-diet-fed mice. Since dietary C. albicans colonizes in the gut, one would expect its excessive abundance in mixed fungus-HFD-fed mice. However, while it synergized its own colonization in the mice feeding on nonobesogenic food, interestingly, dietary C. albicans antagonized its growth in HFD-fed mice. Additionally, it also altered the abundance of other fungi. The abundance of Rhodotorula mucilaginosa, Trichosporon insectorum, Aspergillus ruber, and Rhizopus arrhizus was negatively associated with obesity. Thus, the diet form of C. albicans colonizes in the gut and does not disseminate into the bloodstream to cause infection, but it modulates both bacterial and fungal ecosystems in the gut, and this modification could be one of the key regulatory factors responsible for the differential obesity parameters in different groups of mice used in this study.

Our results revealed alterations in multiple biochemical parameters and hormonal imbalance. HFD-fed mice were with excessive abdominal fat deposits, hyperinsulinemia, glycosuria, ketonuria, proteinuria, and imbalances of metabolic hormones and appetite-regulating neuropeptides. These alterations however seem to be regulated and restored by the gut load of C. albicans in BALB/c mice via feeding. The release of increased leptin but reduced ghrelin hormones by HDF-fed mice suggested an imbalance in food intake versus consumption in these mice, and reversal of this effect was observed in the C. albicans blended diet. Similar to our study, the leptin/ghrelin ratio was found to be significantly higher even in overweight/obese men than in lean individuals (59). Adipokines, in particular leptin, mediate the cross talk between adipose tissue and metabolic organs, especially the liver, muscle, pancreas, and central nervous system, and play a central role in appetite and body weight homeostasis (60), and C. albicans directly or indirectly inhibited excessive leptin and insulin release, although the mechanism involved requires further investigation. However, more ghrelin production helped in increased food intake and body weight gain in mice fed C. albicans mixed with normal diet. In addition to insulin, a recent finding suggested the role of the resistin hormone in an association between obesity and type 2 diabetes, and it represents a potential candidate for the etiology of insulin resistance (34). As reported earlier, we found a higher level of resistin secretion in our DIO animal models, indicating impaired glucose tolerance and insulin action that was mitigated by dietary C. albicans. Amylin is also cosecreted with insulin from the pancreatic β-cells to play a role in glycemic regulation by slowing gastric emptying and promoting satiety, thereby preventing postprandial spikes in blood glucose levels (61). Surprisingly, although the blood random blood sugar (RBS) level was unaltered in various groups of mice, the amylin level was lowered in the diets containing C. albicans.

Current approaches for the treatment of obesity, including diet and lifestyle changes, have not been successful. Improved understanding of the microbiome, including the mycobiome, and their association with obesity may yield new treatment measures for obesity-associated disorders. The transplantation of microbiome isolated from fecal samples of nonobese mice controlling metabolic syndromes in obese mice implicates the role of these microorganisms in obesity and has given a new potential dimension to managing obesity (62). Meanwhile, because ways to culture some of the microorganisms are still unknown; the application of the microbiome to control obesity seems not to be a viable option. The number of uncultured bacterial species in the gut microbiota currently stands at 1,952 (63). Therefore, a successful experimental demonstration of the role of these fungal or bacterial species individually or in a mixture in regulating obesity will be a comprehensive approach with better translational potential. Here, by taking the commensal microbe C. albicans as a model fungus, we demonstrated the beneficial attributes of this microbe on the murine physiology. Mice have an endogenous level of C. albicans in the gut that increases substantially with HFD consumption or addition of it to the normal diet exogenously. However, dietary C. albicans somehow inhibits its excessive colonization when HFD is consumed. Thus, a threshold level of C. albicans colonization in the gut is needed to maintain eubiosis in the gut, where microbes collectively facilitate the burning of excess calories in the high-fat diet and minimize abdominal fat storage to reduce obesity-induced complications. Therefore, we suggest a possible use of C. albicans as a probiotic, especially in obese people or people dependent on high-fat calorie intake to manage obesity and associated complications. Since we observed an abundance of C. albicans as a natural colonizer in the BALB/c mice gut as opposed to C57BL/6 mice and human hosts, to extrapolate these findings, demonstration in humanized mice models will be of high significance (64–66). More importantly, as several genomic knockout strains of C. albicans were shown to be attenuated, such candidate strains may be the preferable probiotic isolates (18, 67). In conclusion, this study is the first to show that C. albicans is not always an adversary but rather can be a bona fide companion of the host.

## MATERIALS AND METHODS

### Animals.

All mouse experiments were conducted in strict accordance with the guidelines of the Institutional Animal Ethical Committee, Institute of Life Sciences, Bhubaneswar, India. Experimental mouse protocols were fully approved by the committee and given the ethical permit no. ILS/IAEC/133-AH/AUG-18. All efforts were made to minimize suffering and ensure the highest ethical and humane standards.

### Chemicals, reagents, and antibodies.

A high-fat diet (no. 260HF; ~36% fat) and corresponding control diet (no. D-131; ~5% fat) with similar composition were procured from SAFE Diets, Augy, France. Yeast extract-peptone-dextroxe (YPD) medium (no. G037-500g), sulfosalicylic acid (SSA) solution (3%), and Benedict’s reagent were from HiMedia, India. The EnzyChrom ketone body assay kit (EKBD-100) was from BioAssay Systems, Hayward, CA, USA. The Dr. Morepen GlucoOne blood glucose monitoring system (model BG-03) and BG-03 test strips were from Morepen Laboratories, Ltd., Delhi, India. The periodic acid-Schiff (PAS) stain kit (mucin stain; no. ab150680) was obtained from Abcam, Cambridge, MA, USA. The Milliplex MAP mouse metabolic hormone magnetic bead panel was purchased from Millipore-Merck, Darmstadt, Germany, and used for the simultaneous quantification of amylin (active), C-peptide 2, ghrelin (active), GIP (total), GLP-1 (active), glucagon, insulin, leptin, MCP1, PP, PYY (total), and resistin.

### Diet-induced obesity and C. albicans in oral diet.

In-house-bred male BALB/c mice about 4 to 5 weeks of age were cohoused and maintained under standard conditions, such as a highly ventilated space with HEPA-filtered air, a maintained environment temperature, clean bedding, and a 12-h/12-h dark/light cycle in individually ventilated cages. The initial body weight of each ear-marked mouse was measured using a precision physical balance, and subsequent weight was measured after every 30 days. Age-, body weight-, and blood glucose-matched mice (4 mice per cage) were divided into different groups. The cages were supplemented with soft food dough (prepared with or without C. albicans in the normal diet [ND] or high-fat diet [HFD]) and water provided *ad libitum* to the respective experimental groups for the duration of 21 to 22 weeks as mentioned elsewhere. Collection of mouse urine and fecal samples was carried out by placing the mice in metabolic cages 24 to 48 h prior to the sacrifice schedule on the 150th day of dietary intervention. On the day of sacrifice, under anesthesia (isoflurane inhalation) mouse blood was collected via cardiac puncture using a 2-mL syringe and 26-gauge needle with potassium EDTA anticoagulant vials. Following immediate centrifugation at 4°C, plasma was separated and stored at −20°C until analysis. Mice were sacrificed by cervical dislocation and dissected for the isolation of kidneys (tissue histology).

### Microbial DNA extraction from mouse fecal sample.

The microbial metagenomic DNA from the mouse fecal samples was extracted by using a well-established guanidinium thiocyanate (GITC)-based protocol with a minor modification (68). Briefly, about 200 mg of stool sample was resuspended in Tris-EDTA buffer (pH 8.0) and homogenized using sterile glass beads (2.5 mm), and the suspension was collected after centrifugation followed by enzymatic lysis using a cocktail of lysozyme (10 mg/mL) (MFCD00131557; Mp Biomedicals India Pvt., Ltd.), Zymolyase (20 mg/mL) (08320921; Mp Biomedicals India, Pvt., Ltd.), and lyticase (20 mg/mL) (37340-57-1; Sigma-Aldrich) at 37°C overnight. The next day, 250 μL of 4 M GITC was added to the suspension, the mixture was vortexed for 1 min, and then 120 μL of 10% *N*-lauryl sarcosine and 650 μL of 5% *N*-lauryl sarcosine were added, respectively, with an interval of 10 min. The mixture was vortexed and incubated at 70°C for 1 h. The mechanical cellular disruption was carried out with 0.1-mm silica beads (1177 P71-Mp Biomedicals India, Pvt., Ltd.) in a FastPrep 24 5G homogenizer (Mp Biomedicals India, Pvt., Ltd.) 3 times with a cycle of 30 s of beating and 30 s at rest followed by washing in 1 mL of polyvinyl polypyrrolidone (PVPP) (77627; Sigma-Aldrich). The supernatant was collected from the homogenized samples by centrifugation, mixed gently with 2 mL isopropanol, and incubated overnight at 4°C. After incubation, crude DNA was precipitated by centrifugation and dried at 42°C for 1 h. The dried pellet was resuspended in 900 μL of phosphate-buffered saline (PBS) and 100 μL of 0.5 M potassium acetate and incubated at −20°C for 1 h, followed by incubation at 4°C for 30 min. RNase A (10 mg/mL) (DS0003; HiMedia) treatment was carried out for 30 min at 37°C. Finally, DNA was extracted by using 96% chilled ethanol and spinning at 14,000 rpm for 30 min at 4°C. The pellet was dried at 42°C for 1 h and suspended in 60 to 100 μL of Tris-EDTA (TE) buffer (pH 8.0) depending upon the pellet amount. The DNA concentration was determined in NanoDrop spectrophotometer with a Qubit dsDNA HS assay kit (Q32854; Invitrogen, USA) and further verified by agarose gel electrophoresis.

### Semiquantitative and real-time PCR detection of C. albicans in fecal DNA.

Isolated total DNA from the fecal samples of the four mice groups were labeled as BND or BCND (BALB/c mice subjected to a normal diet without or with C. albicans mix, respectively) or BHFD or BCHFD (BALB/c mice subjected to a high-fat diet without or with C. albicans mix, respectively). To identify the presence of C. albicans DNA in these samples, semiquantitative endpoint PCR was performed to amplify CaPCNA and CaEfg1. PCR was carried out in 100 μL master mixture containing deoxynucleoside triphosphates (dNTPs) (200 μM), 20 μL 1× Taq-DNA polymerase buffer, 2 U of Taq-DNA polymerase, 20 pmol of CaPCNA (NAP31, 5′-GGC CAA GCT TGG ATC CAC ATA TGT TAG AAG GTA AAT TTG AAG-3′; NAP32, 5′-GGC CGA ATT CGG ATC CCT ACT CAT CAT CAT CG-3′) or CaEFG1 (NAP393, 5′-CCG GGG TAC CGG ATC CAC ATA TGT CAA CGT ATT CTA TAC; NAP395, 5′-GGC CCT CGA GAC CAC TAG GAG CAC TTG TG-3′) primers, and 200 ng of total DNA as a template. The PCR cycling condition included initial denaturation of 1 min at 95°C, followed by 25 cycles of 95°C for 30 s, 52°C for 30 s, and 72°C for 30 s, with a final extension of 3 min. The PCR products were separated on 1% agarose gel, and images were taken with a Bio-Rad ChemiDoc MP imager system. To estimate the abundance of C. albicans in various groups of fecal samples, real-time PCR (RT-PCR) was performed. The RT-PCR was performed in QuantStudio 3 real-time PCR system using Fast SYBR green master mix with CaPCNA (NAP212, 5′-GGT GGT GGA GGA GAT TCT GTC AAG TTT-3′–NAP32) and CaEFG1 (NAP819, 5′-CCA GGG TGG TGC TGC TAA TAG-3′; NAP820, 5′-GGG TGA AGG GTG AAC TGA ACC-3′) primers and 50 ng of microbial DNA. The fast cycle condition was 95°C for 2 min of initial denaturation, followed by 40 cycles of 95°C for 5 s and 60°C for 30 s. The *C_T_* values were used to generate a bar graph. The experiment was repeated with experimental duplicates.

### CFU analysis.

Fungal colonization was estimated in the fecal samples by CFU determination. About 200 mg of each of the samples from various groups was separately homogenized in sterile PBS, diluted to various concentrations, and further spread onto YPD agar containing chloramphenicol (25 mg/mL) to observe various fungal colonies. Even at the maximum dilution, most of each plate was covered with molds, and only 10 to 30 isolated colonies could be seen. Random isolated colonies were picked, and colony PCR was conducted to confirm the presence of C. albicans species. PCR amplification was carried out for the CaPCNA gene.

### Metagenomics analyses.

The composition of bacterial and fungal species in the gut of various groups of mice was determined by 16S and ITS metagenomics analyses of fecal DNA samples. After passing quality control, libraries were constructed in alignment with the 16S metagenomic library preparation protocol for bacteria and archaea from Illumina, Inc. Briefly, 12.5 ng DNA was subjected to 16S V3-V4 PCR using the gene-specific primers Fp (5′-*TCG TCG GCA GCG TCA GAT GTG TAT AAG AGA CAG* CCT ACG GGN GGC WGC AG-3′) and Rp (5′-*GTC TCG TGG GCT CGG AGA TGT GTA TAA GAG ACA G*GA CTA CHV GGG TAT CTA ATC C-3′), which target the V3 and V4 regions of 16S rDNA (69). Similarly, for the ITS analyses, the “Fungal Metagenomic Sequencing Demonstrated Protocol” from Illumina was used. The gene-specific sequences used in this protocol target the fungal ITS1 region between the 18S and 5.8S rRNA genes (27). Briefly, 12.5 ng DNA was subjected to PCR using ITS1F/1R (5′-*TCG TCG GCA GCG TCA GAT GTG TAT AAG AGA CAG* CTT GGT CAT TTA GAG GAA GTA A-3′ and 5′-*GTC TCG TGG GCT CGG AGA TGT GTA TAA GAG ACA G*GC TGC GTT CTT CAT CGA TGC-3′) and ITS4F/4R (5′-*TCG TCG GCA GCG TCA GAT GTG TAT AAG AGA CAG* CCC GGT CAT TTA GAG GAA GTA A-3′ and 5′-*GTC TCG TGG GCT CGG AGA TGT GTA TAA GAG ACA G*GC TGC GTT CTT CAT CGA TGT-3′) primers. The overhang adapter sequences of these primers are italicized.

Both of the PCR products from all the four cohorts were bead purified and subjected to another round of PCR with dual indices and adapters to generate the respective libraries as recommended by Illumina. The cleaned libraries were quantitated on a Qubit fluorometer, and appropriate dilutions were loaded on a D1000 screen tape to determine the size range of the fragments and the average library size. Libraries were diluted to 4 nM, pooled, spiked with a 20% PhiX premade library from Illumina, and loaded on a MiSeq v.3 kit. Sequencing was performed for 2× 300 cycles. The original raw data obtained from Illumina Miseq were recorded in FASTQ files. The reads obtained from the instrument were adapter free. The paired-end reads were assembled, filtered, trimmed, and aligned to the SILVA 16S database using Mothur software v.1.44.1. The reads were clustered based on similarity and further clustered into operational taxonomic units (OTUs) at 97% identity against the Greengenes database (“gg_13_8_99”). For ITS analyses, Qiime2 q2cli v.2021.2.0 was used to analyze the raw reads after removing the low-quality, noisy, unpaired, and chimeric reads by DADA2. The 118 amplicon sequence variants (ASVs) from the analysis were classified using the UNITE dynamic classifier. The relative abundances were calculated using R. Relative abundances of the top 10 to 20 phyla, classes, orders, families, genera, and species were determined. Taxonomic lineages at rank 11 or higher with respect to relative abundance, were collapsed as “others.” The relative abundance (percentage) of each lineage was tabulated at each level. Unidentified categories are the ones for which taxonomy is not presently available. This is different from “unclassified,” which means the reads could not be classified by the classifier.

### Measurement of blood glucose.

On the morning of the 150th day, the tip of the tail of the mouse under anesthesia was cut, and a drop of the blood oozing out was touched to the GlucoOne BG-03 test strip in a GlucoOne blood glucose monitoring system for the measurement of random blood glucose. The displayed values were noted, and glucose was expressed as milligrams per deciliter of blood.

### Estimation of urine sugar.

To 1 mL of Benedict’s qualitative reagent, 100 μL of mouse urine was added, and the reagent and urine were mixed, boiled over a flame for 5 to 10 min, and then cooled under tap water. The content of the urine turns turbid due to precipitates that range from green to red, depending on the increasing amounts of reducing sugar (70). The formed turbidity of precipitate was measured at 650 nm using an Eppendorf BioPhotometer Plus.

### Urine protein estimation.

Urinary total proteins were measured by precipitating the proteins with 3% sulfosalicylic acid (SSA). The turbidity thus formed was compared with that of the protein standard bovine serum albumin at 650 nm. One part of urine or standard (1:2 serially diluted concentration with 10 mg/mL as maximum and 0.3125 mg/mL as minimum) was gently mixed with 4 parts of 3% SSA, and after 5 min at room temperature (25 to 32°C), the absorbance of assay mixture was measured at 650 nm and 420 nm against the reagent blank using a VICTOR Nivo multimode microplate reader or Eppendorf BioPhotometer Plus in different experiments. A known concentration of protein solution was used to prepare a standard curve in the range from 0 to 10 mg/mL. The unknown urinary protein concentration was then determined from a plot of concentration versus absorbance obtained for the standard protein solutions (71).

### Histopathology.

Mouse kidney paraffin sections were stained with periodic acid-Schiff reagents and counterstained with Mayer’s hematoxylin as per the manufacturer's instructions (18).

### Ketone body analysis.

Concentrations of acetoacetic acid (AcoAc) and 3-hydroxybutyric acid (BOH) were measured in plasma or urine samples from mice with the EnzyChrom ketone body assay kit as per the manufacturer’s instructions. The assay is based on 3-hydroxybutyrate dehydrogenase catalyzed reactions, in which the change in NADH absorbance at 340 nm is directly related to the AcoAc and BOH concentrations. At pH 7.0, the reaction is AcAc + NADH + H+ ⇋ BOH + NAD+; the reverse reaction happens at pH 9.5 and is AcAc + NADH + H+ ⇋ BOH + NAD+. As per the assay guide, the concentration of AcoAc = (OD of blank – OD of sample)/(OD of H_2_O – OD of standard) × 8 and the concentration BOH = (OD of sample – OD of blank)/(OD of standard – OD of H_2_O) × 8 were determined and were expressed as the millimolar concentration of the respective ketones. The total ketone body (TKB) concentration was calculated as TKB = (AcoAc +BOH) and expressed as the millimolar concentration.

### Mouse hormone assay.

Leptin, ghrelin, resistin, amylin, gastric inhibitory polypeptide (GIP), glucagon-like peptide-1 (GLP-1), pancreatic polypeptide (PP), insulin, glucagon, C-peptide, and peptide tyrosine tyrosine (PYY) were measured from plasma using a mouse multiplex magnetic bead-based metabolic hormone panel as per the manufacturer’s protocol. The samples, standard, control, and blanks in the panel were acquired in the Bio-Plex 200 system and analyzed using 5PL algorithms in Bio-Plex Manager 5.0 software.

### Statistical analysis.

Statistical analysis of data sets derived from multiplex hormone panel and other quantification parameters were carried out using GraphPad Prism 8.0 and Microsoft Excel. Statistical significance was calculated based on two-way or one-way analysis of variance (ANOVA), with Tukey’s *post hoc* multiple-comparison test. Correlation and regression analyses was carried out using Pearson’s correlation tests using GraphPad Prism 8.0.

### Data availability.

The data that support the findings of this study are available from the corresponding author upon reasonable request. Metagenomics data and bacterial and fungal DNA sequencing data have been uploaded to NCBI under BioProject no. PRJNA765929.
