# Supplementary material for: Commensal Fungus Candida albicans Maintains a Long-Term Mutualistic Relationship with the Host To Modulate Gut Microbiota and Metabolism
Source: Microbiol Spectr. 2022 Sep 22;10(5):e02462-22. doi: 10.1128/spectrum.02462-22 (PMC9603587; doi:10.1128/spectrum.02462-22)
Supplement: Supplemental file 1 — Fig. S1 to S6; Tables S1 to S3. Download spectrum.02462-22-s0001.pdf, PDF file, 1.9 MB [file spectrum.02462-22-s0001.pdf]

Supplementary Informations

Commensal fungus *Candida albicans* maintains a long-term mutualistic relationship with the host to modulates gut microbiota and metabolism

Doureradjou Peroumal[1+], Satya Ranjan Sahu[1, 2+], Premlata Kumari[1, 2], Bhabasha Utkalaja[1, 2], and Narottam Acharya[1,*]

[1]*Laboratory of Genomic Instability and Diseases, Department of Infectious Disease Biology, Institute of Life Sciences, Bhubaneswar - 751023, India*

[2]*Regional Centre for Biotechnology, Faridabad, India.*

***Correspondence to:**

Narottam Acharya, Phone: 91-674-2304278, Fax: 91-674-230 0728

E-mail: narottam_acharya@ils.res.in, narottam74@gmail.com

+contributed equally

Running title: Role of *C. albicans* in obesity

Keywords: Obesity, Diet, immune response, metagenomics, microbiome, BMI, hormone, immunity, cytokines, adipokines, probiotics

Abbreviations: HFD: high fat diet; ND: Normal diet; GLP-1: glucagon-like peptide-1; GIP: Glucose-dependent insulinotropic polypeptide; PYY: peptide tyrosine tyrosine; PP: pancreatic polypeptide; DIO: Diet induced obesity; RBG: Random blood glucose;

Supplementary Figure 1: Effect of dietary *C. albicans* on DOI induced body weight and distal gut flora (A) A kinetics of body weight gain with respect to duration from mice fed with or without *C. albicans* in normal diet (ND) or high fat diet (HFD). (**B**) Real-time PCR amplification of CaPCNA and CaEfg1 orfs from the meta-genomic DNA isolated from fecal samples. The average Ct values of 4 sets of data were plotted. **(C)** A representative set of YPD agar + chloramphenicol plates showing the presence of various fungal colonies in the fecal samples of all four groups of mice. Since the fecal sample also contains species like *Aspergillus*, most of the portion of the plates were covered with molds and very few isolated colonies were seen. Random isolated colonies were picked and colony PCR was conducted to confirm the presence of *C. albicans* species.

Supplementary Figure 2: Metagenomics analyses. Various workflow followed in the metagenomic analyses of fecal samples (**A**) 16S rDNA and (**B**) 18S rDNA ITS1 analyses

Supplementary Figure 3: 16s rDNA sequence analyses. (A) Alpha rarefaction plot generated using Simpson to measure average bacterial species diversity within a sample. Taxonomic classification of OTUs was carried out and assigned them into top 20 classes (**B**), top 20 orders (**C**) and top 20 families (**D**). BND, metagenomic DNA isolated from normal diet fed BALB/c mice fecal sample; BCND, metagenomic DNA isolated from normal diet with *C. albicans* mix fed BALB/c mice fecal sample; BHFD, metagenomic DNA isolated from high fat diet fed BALB/c mice fecal sample; and BCHFD, metagenomic DNA isolated from normal diet with *C. albicans* mix fed BALB/c mice groups fecal sample.

Supplementary Figure 4: ITS sequence analyses. (A) Alpha rarefaction plot generated using Simpson to measure average fungal species diversity within a sample. Taxonomic classification of OTUs was carried out and assigned them into top 20 classes (**B**), top 20 orders (**C**) and top 20 families (**D**). BND, metagenomic DNA isolated from normal diet fed BALB/c mice fecal sample; BCND, metagenomic DNA isolated from normal diet with *C. albicans* mix fed BALB/c mice fecal sample; BHFD, metagenomic DNA isolated from high fat diet fed BALB/c mice fecal sample; and BCHFD, metagenomic DNA isolated from normal diet with *C. albicans* mix fed BALB/c mice groups fecal sample.

Supplementary Figure 5: Mean and standard error mean of various metabolic hormones from each of the groups of mice blood on the 150th day (~22 weeks) of dietary intervention, (**A**) plasma leptin to ghrelin ratio, (**B**) peptide tyrosine tyrosine (PYY) (pg/mL), (**C**) Amylin (pg/mL), and (**D**) Glucagon level (pg/mL). A linear regression and Person's correlation analysis of Insulin level versus leptin level from individual mouse irrespective of dietary intervention (**E**). A pie chart depicting different percentage of various metabolic hormones measured from various groups of mice (**F**). A statistical significance (*$p \leq 0.05$, **

$p \leq 0.01$,***$p \leq 0.001$, **** $p \leq 0.0001$) was calculated using one-way ANOVA and Tukey's multiple comparison test.

Supplementary Figure 6: Structure and function of kidney upon dietary manipulation. (**A**) A representative image with table depicts urine glucose level by a semi quantitative Benedict's test. (**B**) Mean and standard error of mean of a kidney weight (gram) from all individual mice from the 4 groups of mice. (**C**) A representative images of kidney longitudinal section (2 micron thickness) from all 4 groups of mice were stained with periodic acid schiff's, counter stained with mayer's hematoxylin and analyzed using brightfield 40x objective, ZEISS ApoTome Microscope. A statistical significance (*$p \leq 0.05$, ** $p \leq 0.01$, ***$p \leq 0.001$, **** $p \leq 0.0001$) was calculated using one-way ANOVA and Tukey's multiple comparison test.

Supplementary Table 1: Kinetics of body weight gain in BALB/c mice. Individual mouse were ear marked and tracked for change in body weight as per the mentioned duration upon diet challenge and presence or absence of *C. albicans* mix.

Supplementary Table 2: Validity of metagenomics. Read summary obtained from Illumina sequencing for each sample.

Supplementary Table 3: Diversity and abundance of bacteria and fungi in the mice gut. Percent abundance of top 20 bacterial and fungal species.

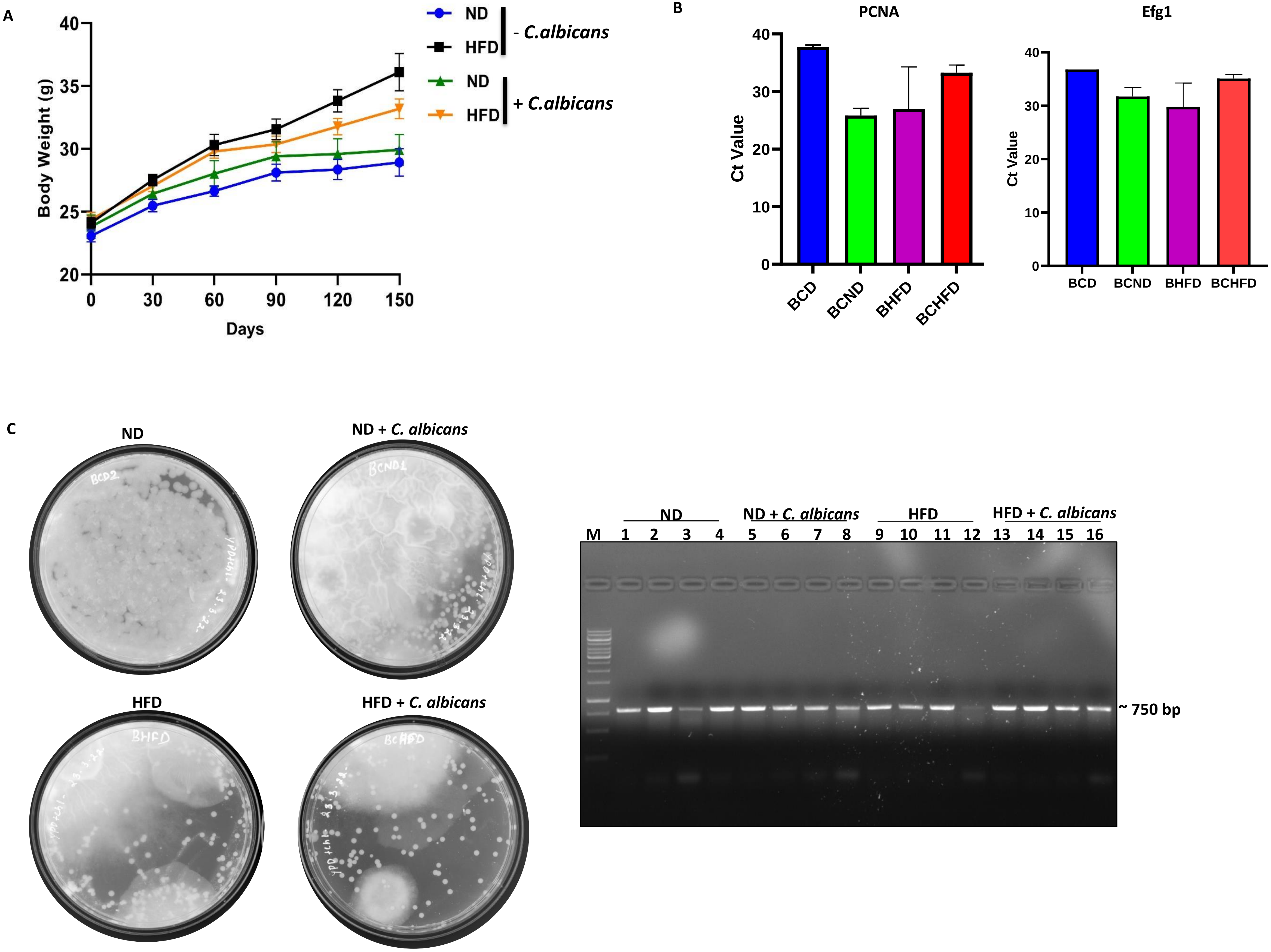

Supplementary Fig.1

A. Workflow for 16S rDNA Analyses

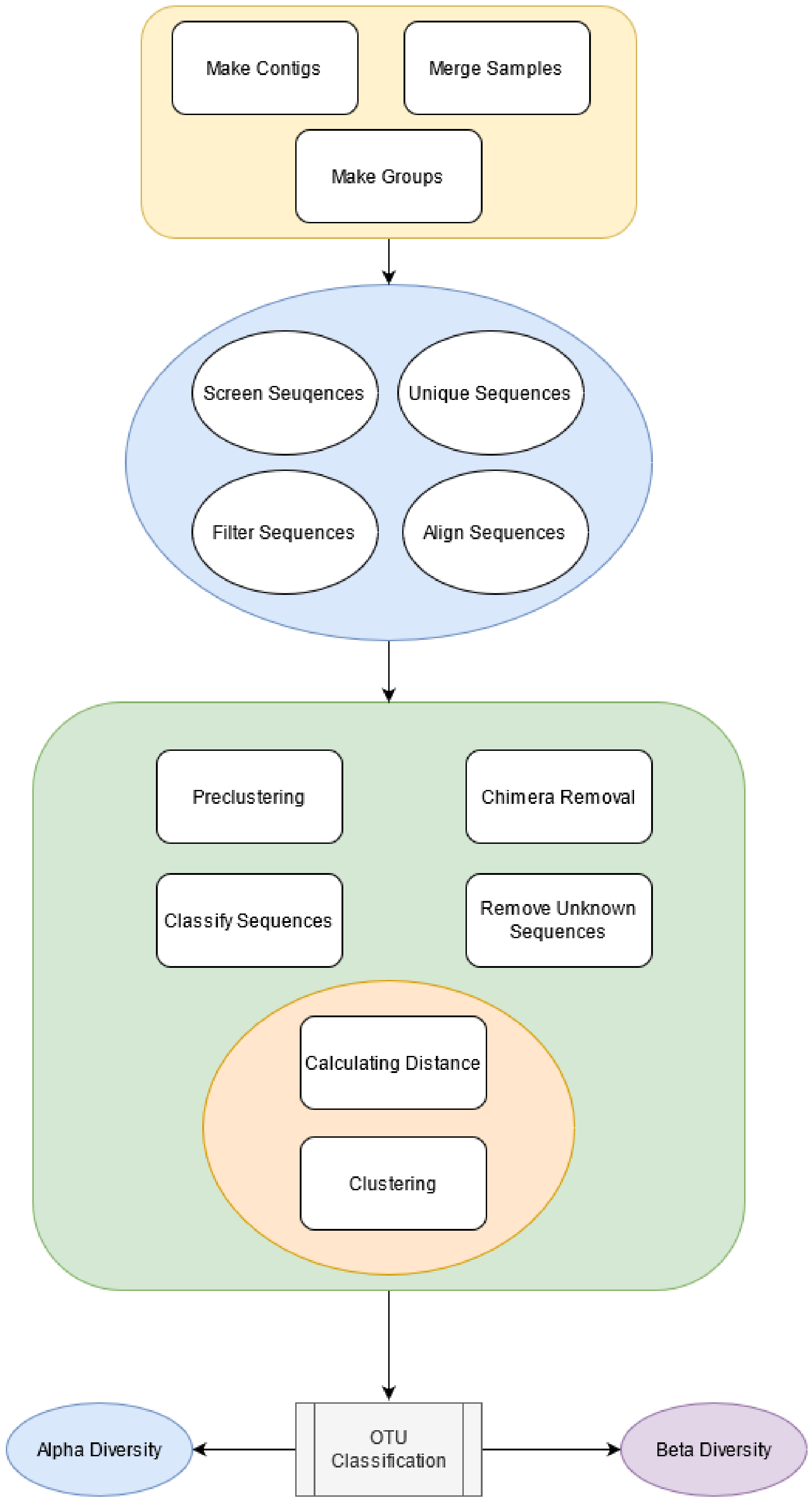

B. Workflow for ITS Analyses

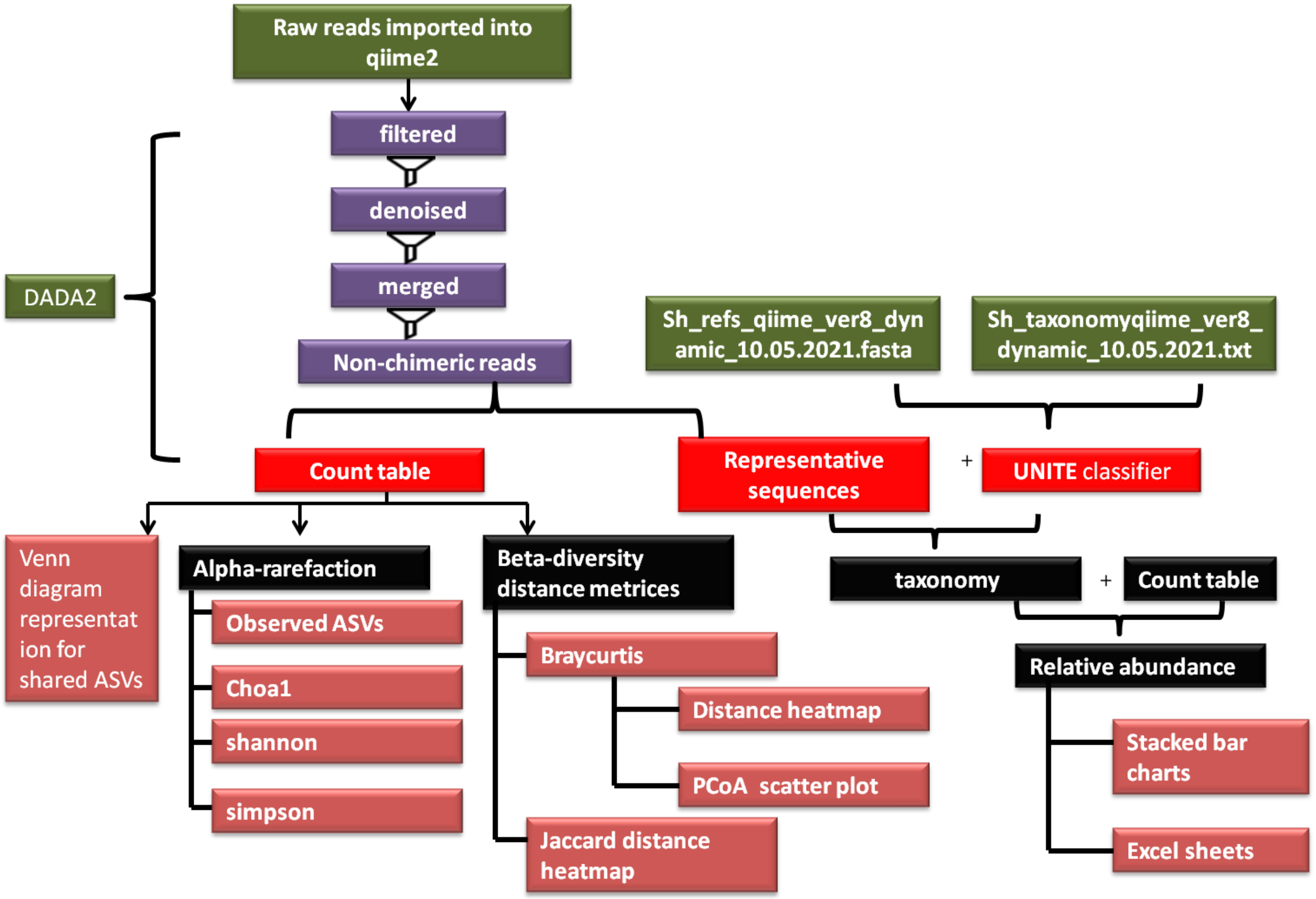

Supplementary Fig. 2

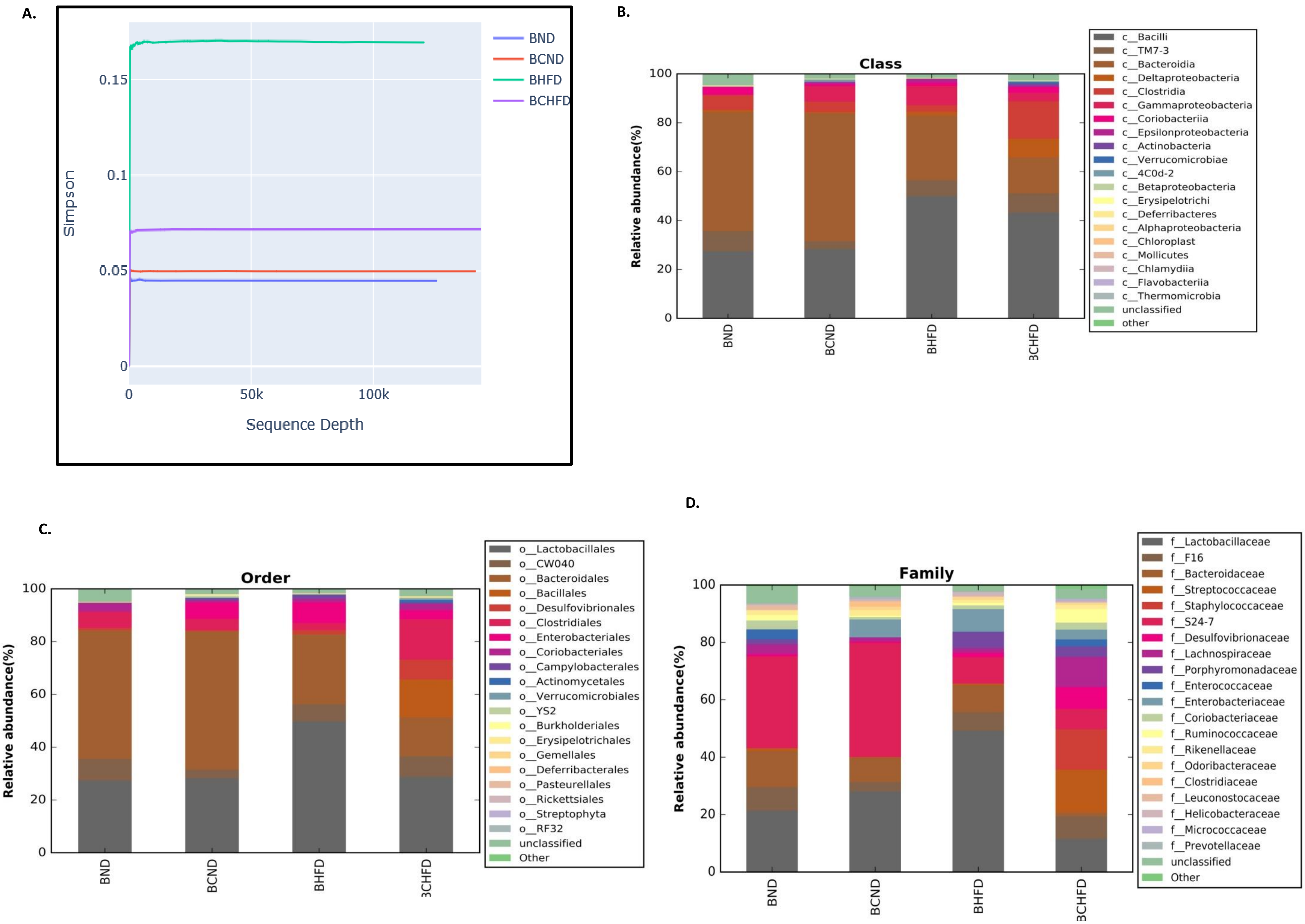

Suppl. Figure 3

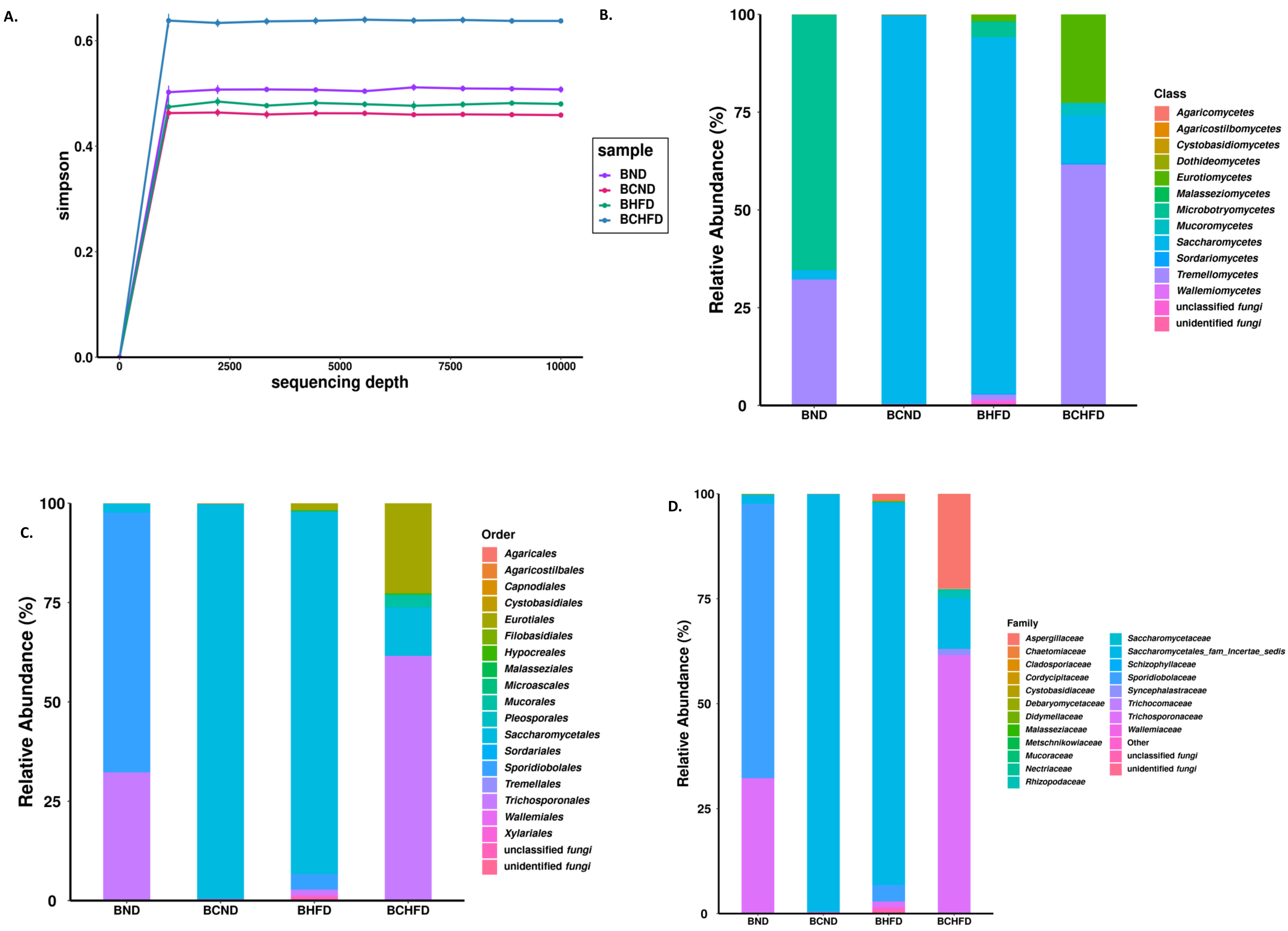

Suppl. Figure 4

A.

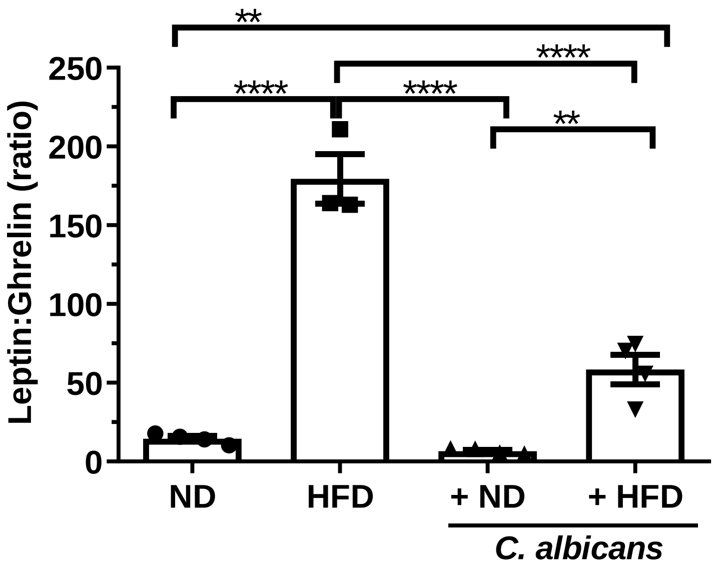

B.

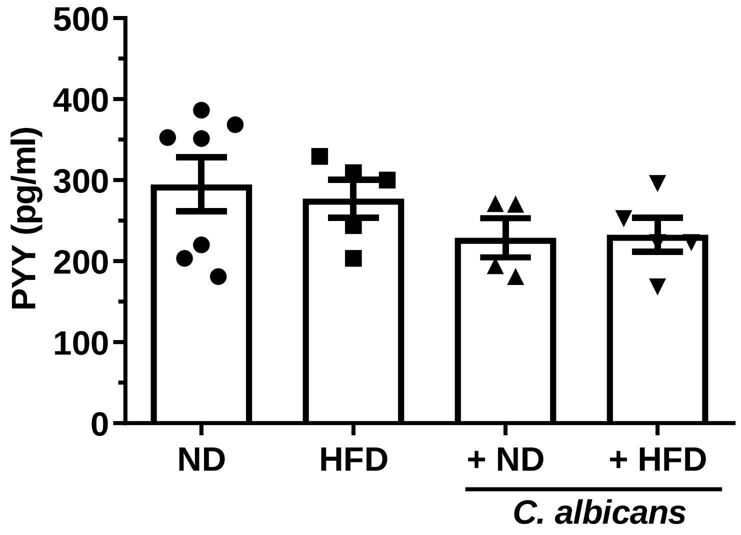

C.

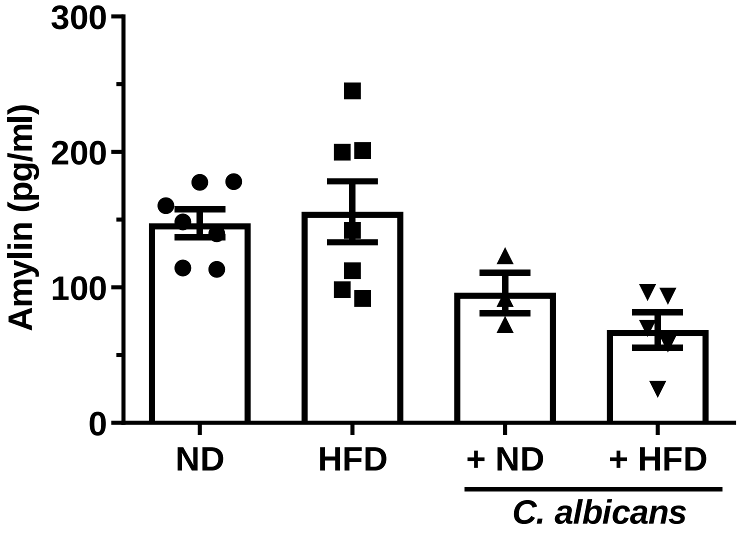

D.

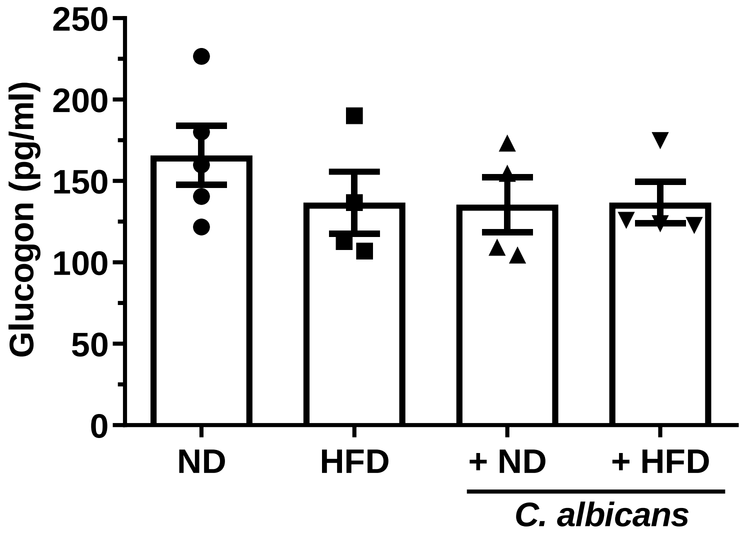

Supplementary Fig.5

E.

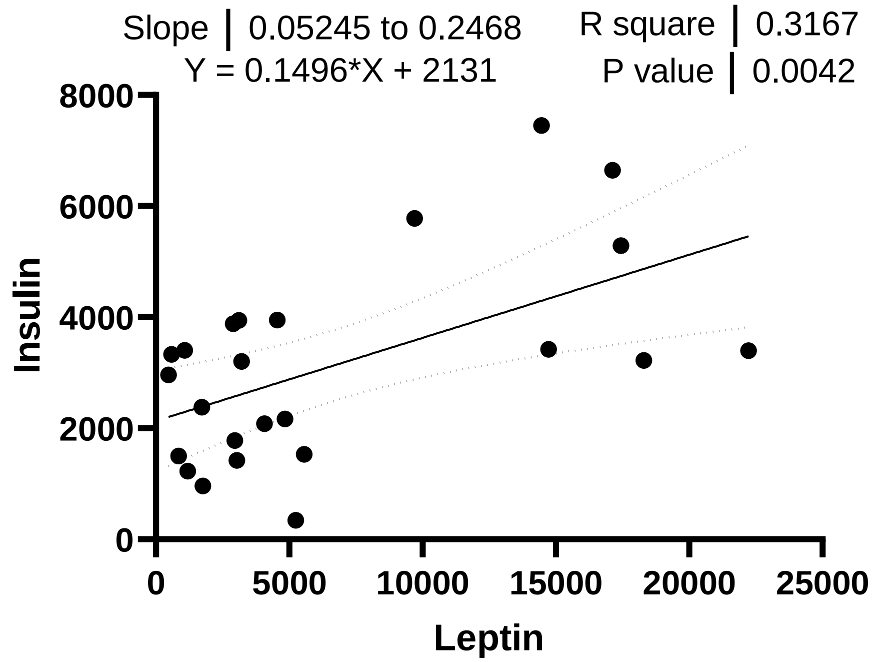

F.

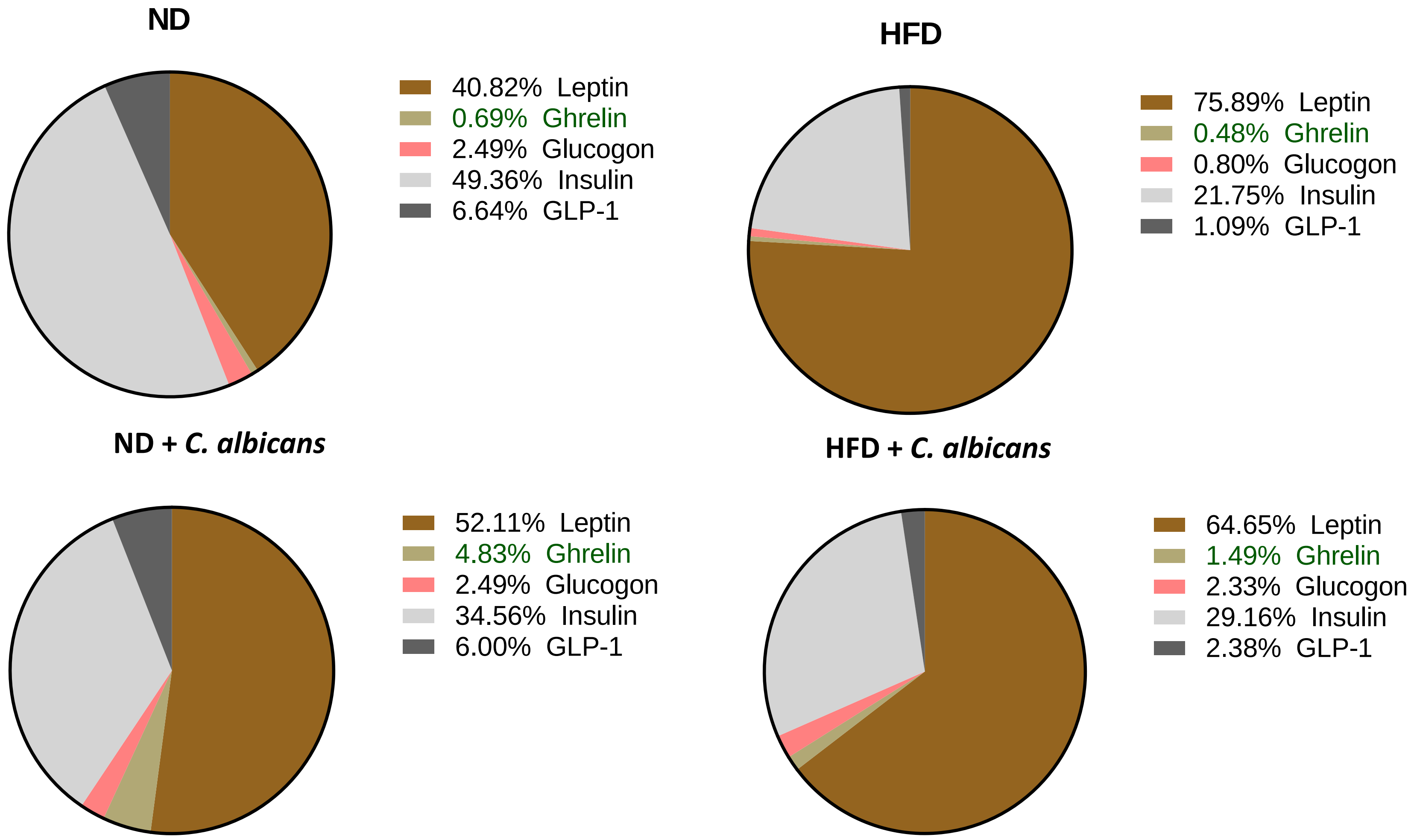

Supplementary Fig.5

A.

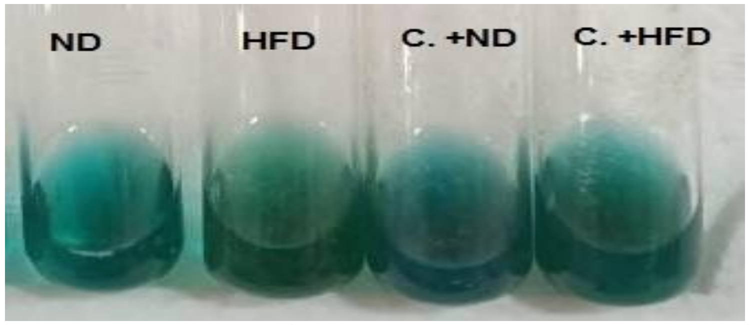

Urine of Diet Conditions	Benedict's Semi quantitative read out
ND	nil (No color change)
HFD	+ (Green)
ND+ C. albicans	Trace/nil (Bluish Green)
HFD+ C.albicans	Trace/nil (Bluish Green)

B.

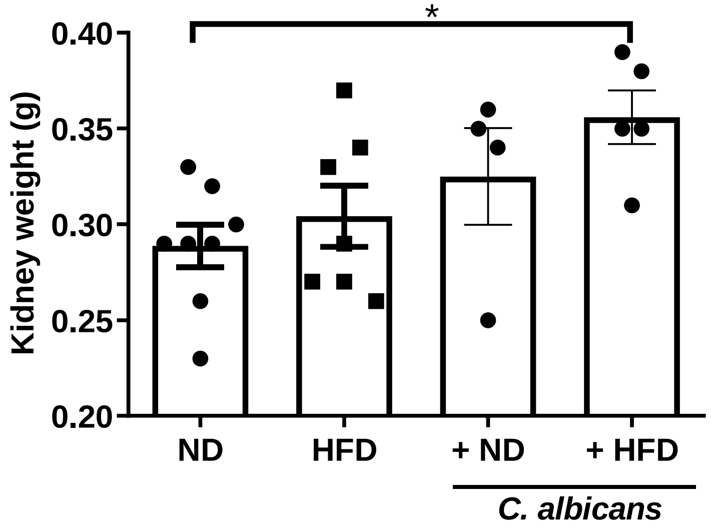

C.

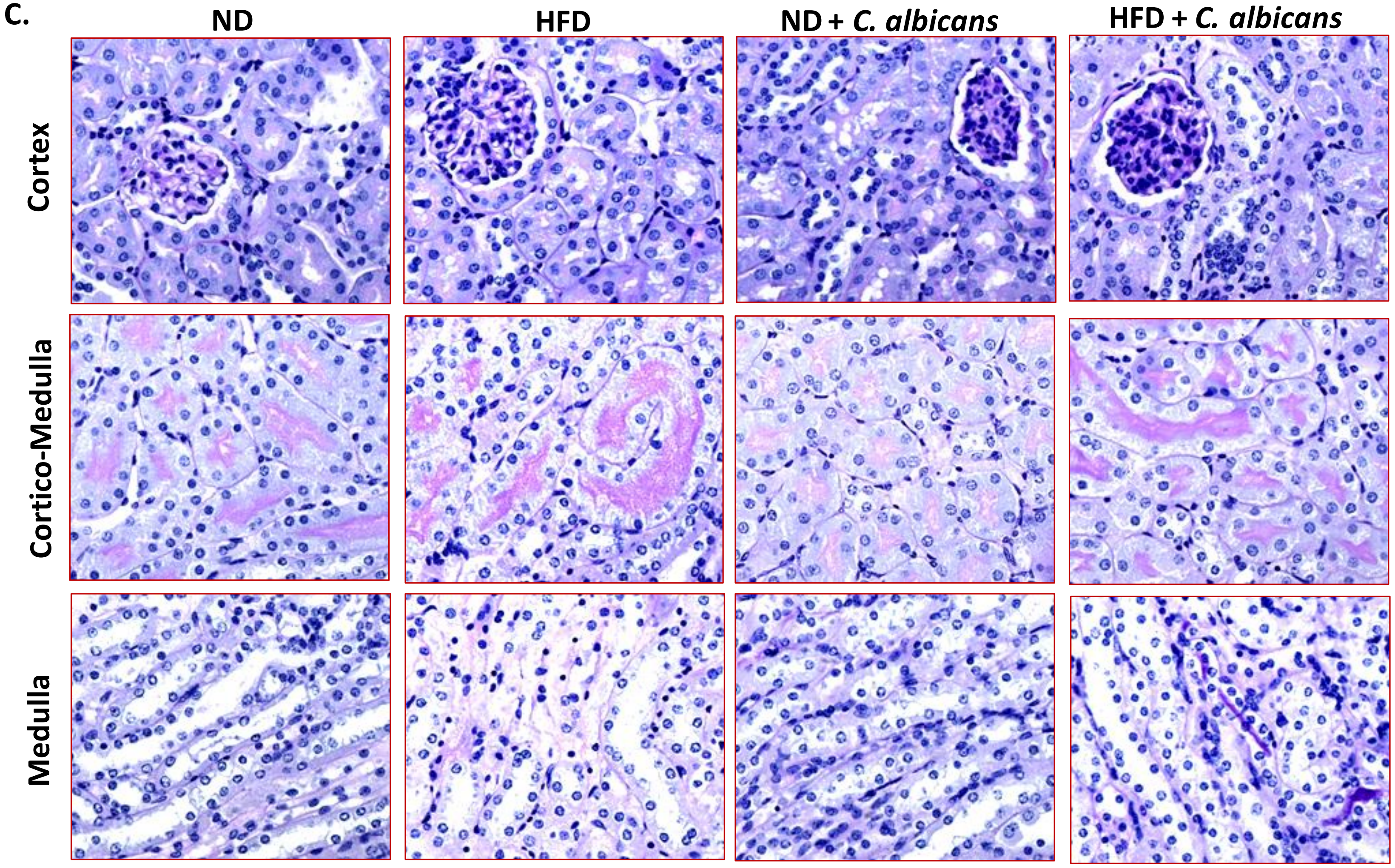

Supplementary Fig. 6

Supplementary Table 1: Individual mouse were ear marked and tracked for change in body weight and blood glucose

Body Wt(g)	Normal Diet						High Fat Diet						RBG (mg/dL)	ND		HFD	
**DAYS**	0	30	60	90	120	150	0	30	60	90	120	150	**DAYS**	0	150	0	150
**Without *Candida albicans* in diet**	25.1	27.8	29	32.1	33.4	36.2	24.2	28.6	30.7	30.6	34.1	35.9	**Without *Candida albicans* in diet**	144	192	119	203
	24.9	27.2	27.6	29.6	29.7	29.5	24.6	26.6	26.8	27.4	29.3	31.1		127	126	132	192
	23	25.3	26	26.7	26.7	26.9	23.6	28	34.5	34.6	38	45		135	187	144	181
	22.5	25.5	26.4	27	27.2	27.9	25.8	27.1	32.4	33.9	33.6	33.8		163	166	142	203
	21.2	24.2	25	26.9	27	27.3	24.2	26.9	28.2	31	31.8	32.6		161	195	139	154
	22.1	24.2	26.1	27.3	27.5	27.5	24.4	26.6	30.8	32.7	35.1	36.4		117	180	158	164
	23	25	26.4	26.9	26.7	27.1	24.6	30	30	32.4	34.6	37.1		153	173	165	183
	22.8	24.6	26.7	28.3	28.7	29	21.5	26.4	29.1	29.9	34.1	37		163	168	167	163
**Mean**	23.1	25.5	26.6	28.1	28.4	28.9	24.1	27.5	30.3	31.6	33.8	36.1	**Mean**	145.4	170.8	145.8	177
**SEM**	0.5	0.5	0.4	0.7	0.8	1.1	0.4	0.4	0.9	0.8	0.9	1.5	**SEM**	16.49	20.53	15.57	17.44
**Weight gain (%)**	0	10.39	15.15	21.64	23	25.1	0	14.1	25.72	31.1	40.2	49.8					
**With *Candida albicans* in diet**	22.4	24.7	26.3	27.2	27.3	27	23.4	27.1	27.9	29	30.2	30.4	**With *Candida albicans* in diet**	156	208	185	180
	25.7	29.1	29	30.3	30.1	29.6	22.2	24.7	29	29.5	29.8	31.5		171	149	143	155
	22	24.6	26	27.3	27.4	28	23.3	26.7	29.9	28.2	31.3	32.3		139	149	128	194
	19.6	22.3	24.1	25.8	25.9	26.2	23.3	26.6	28.7	30.6	33.6	36.4		151	159	157	174
	26.4	29.5	32	34.8	35.4	35.2	24.5	28.2	30.8	31.4	32.3	34.3		184	145	153	168
	25.5	27.1	30.3	31.4	31.7	32.8	26.1	28.3	31	32.9	33.3	34.9		147	152	171	163
	25	27.7	28.5	29.1	29.3	30.7	26.5	28.3	32.4	32.8	34.2	35		134	146	139	173
	22.3	dead	dead	dead	dead	dead	25.8	26.4	28.6	28.5	29.5	30.7		142	died	140	159
**Mean**	23.8	26.4	28	29.4	29.6	29.9	24.4	27	29.8	30.4	31.8	33.1	**Mean**	153	158.3	152	172.4
**SEM**	0.9	1	1	1.2	1.2	1.2	0.6	0.4	0.5	0.7	0.6	1.2	**SEM**	15.86	20.74	16.49	11.7
**Weight gain (%)**	0	10.9	17.64	23.5	24.4	25.6	0	10.6	22.1	24.6	30.3	35.6					

Supplementary Table 2: Quality check summary of 16S RNA and ITS sequence analyses

Samples	Read length (bp)	Raw reads	Total reads in bp	Reads after filtration	#OTU /#ASV	Alpha Diversity Indices (Simpson)
**16S RNA Analyses**						
**BND**	35-301	272568	163540800	126118	1500	0.044
**BCND**	35-301	324926	194955600	141864	1628	0.049
**BHFD**	35-301	249693	149815800	120919	1263	0.167
**BCHFD**	35-301	340325	204195000	144057	1695	0.071
**ITS 18S RNA Analyses**						
**BND**	35-301	243714	146228400	61774	22	0.501
**BCND**	35-301	205305	123183000	96481	28	0.457
**BHFD**	35-301	233519	140111400	90977	78	0.485
**BCHFD**	35-301	267408	160444800	74611	25	0.638

Supplementary Table 3: Percent abundance of bacterial and fungal species in each samples

Bacteria	Top 20	Genus/Species	BND	BCND	BHFD	BCHFD
Genus	1	***Lactobacillus***	0.208014566	59.74424552	64.94767575	15.75676749
	2	***Bacteroides***	0.25457188	18.0728148	12.54032302	2.06382536
	3	***Streptococcus***	0.016578556	0.38664059	0.637513806	20.01116093
	4	***Staphylococcus***	0.002044369	0.076726343	0.104976544	19.16652479
	5	***Pediococcus***	0.221175193	0.00752219	0.029524653	0.002837523
	6	***Desulfovibrio***	0.016211209	0.991424703	1.971590723	10.29926414
	7	***Clostridium***	0.004488029	4.054460659	0.115911601	8.299755973
	8	***Parabacteroides***	0.034738305	0.203099142	7.445680106	4.955261714
	9	***Enterococcus***	0.069412723	0	0.104976544	3.254639351
	10	***Escherichia***	1.59716E-05	5.077478562	3.993482706	0.001891682
	11	***Adlercreutzia***	0.061634537	1.712050549	1.751796083	3.268826968
	12	***Oscillospira***	0.025187267	0.544606589	1.054139466	6.063787526
	13	***Odoribacter***	0.00253949	2.480818414	1.595424772	1.029075157
	14	***AF12***	0.005590072	0.105310666	0.790604599	1.43105764
	15	***Weissella***	0.033764035	0	0.010935057	0.000945841
	16	***Helicobacter***	0.001309674	0	1.903793371	0.152280423
	17	***Flexispira***	0.003849164	1.499924778	0.062329823	0.472920568
	18	***Rothia***	0.000255546	0.398676094	0.215420617	1.117038382
	19	***Prevotella***	0.008512881	1.594704378	0.008748045	0.001891682
	20	***Akkermansia***	0	0	0	0.84085277
Species	1	***Bacteroides_acidifaciens***	37.04065041	8.487563362	53.05786003	3.785554936
	2	***Pediococcus_acidilactici***	49.18518519	0.035364847	0.138268454	0.020914668
	3	***Desulfovibrio_C21_c20***	0	6.294942827	1.063603489	64.82152817
	4	***Parabacteroides_distasonis***	2.09936766	0.141459389	24.95745586	22.7481874
	5	***Escherichia_coli***	0.003613369	39.78545326	19.4213997	0.013943112
	6	***Clostridium_butyricum***	0	30.81457032	0.015954052	0.013943112
	7	***Weissella_cibaria***	7.551942186	0	0.053180174	0
	8	***Akkermansia_muciniphila***	0	0	0	6.19771333
	9	***Ruminococcus_gnavus***	2.196928636	13.16751149	0.388215273	0.969046291
	10	***Mucispirillum_schaedleri***	1.416440831	0.04715313	0.186130611	0
	11	***Alistipes_indistinctus***	0.256549232	0.011788282	0.116996384	0.104573341
	12	***Butyricicoccus_pullicaecorum***	0.021680217	0.023576565	0.074452244	0.543781372
	13	***Streptococcus_infantis***	0.032520325	0.070729695	0.106360349	0.139431121
	14	***Serratia_marcescens***	0.104787715	0	0.069134227	0.006971556
	15	***Aggregatibacter_pneumotropica***	0	0.459743015	0	0.006971556
	16	***Propionibacterium_acnes***	0.014453478	0.212189084	0.005318017	0.104573341
	17	***Clostridium_methylpentosum***	0.007226739	0	0.159540523	0.125488009
	18	***Kocuria_palustris***	0.003613369	0.023576565	0.005318017	0.055772448
	19	***Jeotgalicoccus_psychrophilus***	0	0	0	0.069715561
	20	Acinetobacter_rhizosphaerae	0	0	0	0.062744004

Fungi	Top 20	Genus/Species	BND	BCND	BHFD	BCHFD
Genus	1	***Aspergillus***	0	0.017663989	0.620482598	22.68181438
	2	***Candida***	2.168346676	99.59165013	91.88702324	12.12444556
	3	***Cystobasidium***	0.07130587	0	0.062270655	0
	4	***Debaryomyces***	0	0.010390582	0	0.046028956
	5	***Fusarium***	0	0.003117175	0.103413766	0.380785003
	6	***Issatchenkia***	0	0	0.064494607	0
	7	***Leptobacillium***	0	0	0.064494607	0
	8	***Malassezia***	0	0.008312466	0.045591015	0
	9	***Meyerozyma***	0.113441156	0	0.02223952	0
	10	***Millerozyma***	0	0	0.041143111	0
	11	***Penicillium***	0	0	0.114533526	0
	12	***Rhizopus***	0	0	0	1.72399364
	13	***Rhodotorula***	65.44096198	0	3.959746469	0
	14	***Saccharomyces***	0	0	0.186811965	0
	15	***Schizophyllum***	0	0.116374518	0	0
	16	***Syncephalastrum***	0	0	0	1.437358775
	17	***Talaromyces***	0	0	0.037807183	0
	18	***Trichosporon***	32.13787962	0.161054021	1.227621483	61.56791363
	19	***Wallemia***	0	0.020781164	0.471477816	0
	20	***Xeromyces***	0	0.058187259	0.879573001	0
Species	1	***Aspergillus_ficuum***	0	0	0	0.226299694
	2	***Aspergillus_ruber***	0	0	0.133450951	45.12538226
	3	***Candida_albicans***	2.284339607	99.78345965	93.41679672	35.37003058
	4	***Candida_hyderabadensis***	0.564165886	0	0	0.073394495
	5	***Cystobasidium_minuta***	0.061738908	0	0.018095044	0
	6	***Hyphopichia_burtonii***	0.014902495	0	0	0.048929664
	7	***Issatchenkia_orientalis***	0	0	0.065594535	0
	8	***Leptobacillium_leptobactrum***	0	0	0.065594535	0
	9	***Malassezia_restricta***	0	0.008328475	0.046368551	0
	10	***Meyerozyma_caribbica***	0.149024951	0	0.022618805	0
	11	***Millerozyma_farinosa***	0	0	0.04184479	0
	12	***Mucor_plumbeus***	0	0	0	0.06116208
	13	***Penicillium_hetheringtonii***	0	0	0.113094026	0
	14	***Rhizopus_arrhizus***	0	0	0	5.039755352
	15	***Rhodotorula_mucilaginosa***	85.9682364	0	4.027278279	0
	16	***Schizophyllum_commune***	0	0.116598651	0	0
	17	***Syncephalastrum_monosporum***	0	0	0	0.159021407
	18	***Trichosporon_insectorum***	10.85540322	0	0.390174391	13.89602446
	19	***Wallemia_tropicalis***	0	0.020821188	0.479518672	0
	20	***Xeromyces_bisporus***	0	0.058299325	0.894573749	0
